# The Contextualized Impact of Ethnic-Racial Socialization on Black and Latino Youth’s Self-Esteem and Ethnic-Racial Identity

**DOI:** 10.3390/bs15111437

**Published:** 2025-10-22

**Authors:** Ashley R. McDonald, Briah A. Glover, Olivia C. Goldstein, Dawn P. Witherspoon

**Affiliations:** 1 T. Denny Sanford Harmony Institute, Arizona State University, P.O. Box 872202, Tempe, AZ 85287, USA; 2 Department of Psychology, The Pennsylvania State University, University Park, PA 16802, USA; bag5694@psu.edu (B.A.G.); ocg5017@psu.edu (O.C.G.); dpw14@psu.edu (D.P.W.)

**Keywords:** ethnic-racial socialization, ethnic-racial identity, self-esteem, neighborhood, parents, adolescents, Black, Latino

## Abstract

This study examined how ethnic-racial socialization (ERS)—cultural-egalitarianism, preparation for bias, and promotion of mistrust—mediate the influence of neighborhood and parental cultural contexts on youth self-esteem and ethnic-racial identity (ERI). Participants included 184 youth (Mage = 13.38; 57.5% female) and 144 parents (Mage = 40.62) from Black and Latino families living in a new destination context. Data were analyzed using multiple group path analysis. Findings revealed distinct patterns for Black and Latino families. Neighborhood disadvantage was negatively associated with preparation for bias and promotion of mistrust beliefs. Neighborhood diversity was positively related to promotion of mistrust, while neighborhood cohesion positively influenced cultural-egalitarianism and preparation for bias beliefs. Each ERS belief was associated with youth perceptions of the corresponding ERS practice. In Latino families, preparation for bias beliefs also supported cultural-egalitarianism practices. ERS practices were linked to youth outcomes. Cultural-egalitarianism was positively associated with self-esteem and, for Latino youth, with centrality and private regard. In contrast, preparation for bias and promotion of mistrust were negatively associated with self-esteem and public regard. Additionally, neighborhood factors, parental discrimination, parental ERI, and ERS beliefs were directly linked to youth self-esteem and ERI. Findings underscore how broader sociocultural contexts shape ERS and, in turn, adolescent development.

## 1. Introduction

Knowledge on the processes by which Black and Latino families develop their youth’s self-system (self-esteem and ethnic-racial identity) has grown exponentially in the past decade (Branje et al., 2021; Umaña-Taylor & Hill, 2020; White et al., 2021). With youth of color comprising at least half of the United States’ adolescent population and growing faster than anticipated, research on the processes by which these families promote positive developmental outcomes is critically necessary (E. P. Smith et al., 2022). Black and Latino families have faced consistent targeted discrimination in the United States based on their race and ethnicity and thus are often siloed in their communities by factors such as redlining (Bloch & Phillips, 2022; Bradford et al., 2024). Ethnic-racial socialization (ERS) is one of the most widely studied cultural parenting practices in Black and Latino families. Research demonstrates that ERS messages work together with neighborhood context to shape youth’s self-system (Anderson et al., 2024; Witherspoon et al., 2021). Ethnic-racial identity (ERI) and self-esteem are critical indicators of positive development in youth of color (D. Hughes et al., 2009a; Rivas-Drake et al., 2014).

Though ERS is common for both Black and Latino families, these families have cultural stressors, assets, beliefs, and practices specific to the history of their ethnic group in the U.S. context. Additionally, research shows these two ethnic-racial groups may differ in how their ERS beliefs and practices are shaped by neighborhood context and parental experiences (Umaña-Taylor & Hill, 2020; Witherspoon et al., 2022). These differences may be particularly pronounced in new immigrant destination areas—communities that have experienced recent and rapid growth in immigrant populations but lack the longstanding infrastructure and support systems of traditional ethnic and immigrant enclaves (Donato et al., 2008; Marrow, 2008).

The complex ways in which Black and Latino families engage in cultural parenting practices in new destination contexts call for analytic models that replicate this complexity. The current study answers this call by meaningfully examining how contextual factors—neighborhood and parental cultural—impact youth’s self-system through a promotive culturally relevant parenting process, ERS. More specifically, the current study explores whether ERS beliefs and practices are the pathways through which neighborhood (i.e., disadvantage, ethnic-racial composition, cohesion, and trust) and parental factors (i.e., perceived discrimination and parent’s ERI) impact adolescents’ self-system (i.e., self-esteem and youth’s ERI). We examine Black and Latino participants separately, as ethnic differences are likely to exist in these processes.

### 1.1. Theoretical Framework

The current study uses the integrative model (García Coll et al., 1996) as a framework for approaching the developmental experiences of youth of color. This model accounts for the unique experiences that families of color have as marginalized people and how these experiences may influence their practices and developmental goals. Stressors rooted in the history of ethnic and racial oppression have resulted in families gaining and passing down cultural assets to protect youth. The integrative model also acknowledges the importance of context when studying processes in families of color. In addition to the integrative model, we use a transactional/ecological perspective which posits that individual processes are interdependent on factors in the microsystem (e.g., families and neighborhoods; D. L. Hughes et al., 2016). This perspective supports our argument that the context in which developmental processes occur is inseparable from the culturally relevant assets and stressors of Black and Latino families. These contexts include the neighborhood, which is affected by structural and interpersonal racism. We focus on objectively measured neighborhood disadvantage and the ethnic-racial composition of the neighborhood, as well as perceived cohesion and trust amongst neighbors, to determine how this context affects youth both directly and through ERS beliefs and practices.

### 1.2. Ethnic-Racial Socialization and the Self-System

Ethnic-racial socialization (ERS) is a process whereby socializers (e.g., parents) provide messages about race and ethnicity to the receiver (e.g., children) in efforts to buffer the effects of widespread racist beliefs and practices (D. Hughes et al., 2009a). The most studied ERS process is between parents and their children. There are four types of ERS: cultural socialization, preparation for bias, egalitarianism, and promotion of mistrust (D. Hughes et al., 2006; Huguley et al., 2019). The majority of work on ERS focuses on cultural socialization and preparation for bias (Umaña-Taylor & Hill, 2020). Cultural socialization messages encourage youth to embrace their race and/or ethnicity by promoting ethnic-racial pride and teaching youth about their ethnic-racial heritage. Preparation for bias messages prepare youth for instances of ethnic-racial discrimination. These messages often include messages about how to cope with this discrimination. Egalitarian messages place value on individual qualities rather than the ethnic-racial group to which one belongs or may suggest that race or ethnicity is not important. Promotion of mistrust messages discourage positive interracial interactions by suggesting wariness of a particular ethnic-racial group.

ERS promotes positive self-system development, particularly in the face of discrimination (Anderson et al., 2024; D. Hughes et al., 2006; Rodriguez et al., 2009; White et al., 2021). We operationalize the youth’s self-system by their self-esteem and ethnic-racial identity (ERI). Adolescents’ self-esteem, or the positive beliefs and confidence one has in themselves, is shaped by the social and environmental effects of racism (García Coll et al., 1996), and protects against risky behaviors (Jackman & MacPhee, 2017). ERI includes the public and private regard of one’s race and ethnicity as well as its centrality to their self-concept (Rivas-Drake et al., 2014). Whereas public regard refers to how one believes others view their ethnic-racial group, private regard is how one views their own ethnic-racial group. Centrality refers to how important one’s ethnic-racial group is to their identity. It is important to include these aspects of the self-system when studying ERS messages because self-esteem and ERI mediate the relationship between ERS and various developmental outcomes (D. Hughes et al., 2009a; Murry et al., 2009; Umaña-Taylor & Hill, 2020).

Differences in these associations are evident when exploring specific ERS types. In their systematic review of ERS in families of color, Umaña-Taylor and Hill (2020) found cultural socialization messages to be consistently linked to positive development of the self, including ERI. Studies with preparation for bias showed mixed results, with its associations with positive developmental outcomes, such as self-esteem, being particularly dependent on the environment and family context. Neighborhood (i.e., structure, disadvantage, ethnic-racial diversity, cohesion, and trust) and parent characteristics (i.e., ERI and ethnic-racial discrimination experiences) were particularly salient, as both could impact ERS messages (D. Hughes et al., 2006; Witherspoon et al., 2023). Interestingly, the review highlighted how objective measures of neighborhood characteristics (e.g., structure and ethnic-racial diversity) were associated with parents’ ERS messages more consistently than participants’ perceptions of these characteristics. Though the literature on egalitarianism and promotion of mistrust is not as extensive, the review reported emerging trends: (1) a positive link between egalitarianism and adjustment and (2) a negative link between promotion of mistrust and adjustment (Umaña-Taylor & Hill, 2020). More work is needed with diverse samples to move the field towards drawing reliable conclusions about these messages.

In recent years, empirical evidence has continued to grow, showing links between Black and Latino parents’ ERS messages and their adolescents’ ERI and self-esteem. Multiple studies show that parental ERS is positively associated with ERI beliefs for both ethnic-racial groups (Martinez-Fuentes et al., 2021; Takamasa et al., 2024). In a meta-analysis on the association between ERS and ERI, ERS was positively associated with Black and Latino youth’s private regard and centrality while negatively associating with their public regard (Huguley et al., 2019). Christophe et al. (2024) found that cultural socialization and preparation for bias were associated with a strong ERI (high centrality and high private regard) in a diverse sample of marginalized adolescents (42.2% Black, 25.9% Latino). In a longitudinal study of Mexican-origin youth, cultural socialization was found to be continually protective for their ERI while facing discrimination throughout adolescence (Stein et al., 2023). For Black youth, cultural socialization was associated with higher self-esteem (Davis et al., 2017; Harris-Britt et al., 2007; D. Hughes et al., 2009b), and preparation for bias was associated with lower self-esteem (Cooper & McLoyd, 2011). More recent work examining ERS’ association with self-esteem has been done with Latino adolescents. Salcido and Stein (2024) found that cultural socialization was positively associated with self-esteem, whereas preparation for bias was negatively associated with self-esteem. The latter relation was no longer found after adjusting for ethnic-racial discrimination experiences. These findings suggest there is more to be uncovered about the effects of preparation for bias messages on Black and Latino youth’s self-system, especially understanding under what conditions these ERS messages are protective. We aim to add to our understanding of how ERS affects adolescents’ self-system developmental processes in the current study.

### 1.3. Parental ERS Beliefs and Practices

An important distinction in knowledge on ERS processes is between parents’ ERS beliefs and ERS practices. Youth’s ERI may be shaped by parents’ beliefs about ERS (Witherspoon et al., 2021). Parents’ ERS beliefs are influenced by the cultural context and history of their ethnic-racial group in the United States as well as the environmental context (D. Hughes et al., 2006; Umaña-Taylor & Hill, 2020). Measuring ERS beliefs with parents’ reports and ERS practices with youth reports allows us to determine how parents’ ERS beliefs map onto the ERS perceived or experienced by the youth. Parsing apart beliefs and practices also allows us to better analyze gender differences in the ERS process. Peck et al. (2014) found that, despite Black parents reporting more cultural socialization messages to their daughters and preparation for bias messages to their sons, youth reports of ERS messages received showed no gender difference. Awareness of this incongruence in beliefs and practices can help scholars to interpret findings and practitioners to implement parenting programs that reflect this nuance.

### 1.4. Neighborhood, ERS, and Youth Self-System

The neighborhood context has been increasingly considered by scholars studying Black and Latino families’ socialization processes. This context is characterized by García Coll et al. (1996) as potentially a promotive or inhibitive environment for youth outcomes. The social and structural processes parents perceive in their neighborhood may make certain cultural messages more important to relay to their children. Neighborhood disadvantage, ethnic-racial composition, and cohesion have varying impacts on the ERS messages parents choose to employ (Pasco et al., 2021a, 2021b; Varner et al., 2022; Witherspoon et al., 2022). In attempts to fill a gap in knowledge on neighborhood-level predictors of ERS strategies, Witherspoon et al. (2022) found neighborhood to account for over 20% of variation in cultural socialization and preparation for bias messages in Black and Latino families. To better understand the culturally protective practices of minoritized families, we must consider the context in which these practices occur.

Multiple studies have found neighborhood ethnic-racial concentration and diversity to significantly impact ERS beliefs and practices (Maereg et al., 2024). In their review, Umaña-Taylor and Hill (2020) note how the measure of neighborhoods’ ethnic-racial composition may influence study results. While objective measures of neighborhood ethnic-racial composition can be pulled from secondary data such as Census or school records, subjective data focuses on how the participant perceives the ethnic-racial makeup of their neighborhood (Witherspoon et al., 2023). Subjective measures can be helpful when aiming to understand how participants’ perceptions shape beliefs and practices, as well as how their perceptions align with objective measures. Objective measures are beneficial when aiming to understand the structural impacts of factors such as ethnic-racial composition. Witherspoon et al. (2022) found that after adjusting for objective characteristics, ethnic-racially affirming neighborhoods were associated with more cultural socialization and fewer preparation for bias messages. Similarly, Varner et al. (2022) found that Black parents utilized more preparation for bias messages when their neighborhood was predominately White or racially diverse. This finding reflects the risk of racial discrimination Black youth face in non-predominately Black environments. For Mexican adolescents, parental cultural socialization made up for the potential toll of living in a neighborhood with fewer people sharing their ethnicity and/or race on their ethnic identity processes (White et al., 2018). In general, high concentrations of neighbors with similar ethnic-racial identities has been proven to be beneficial for Black and Latino youth’s development of self (Pasco et al., 2021b; Stevenson & Arrington, 2009). The current study explores the mediational effect of ERS in this developmental process.

The level of cohesion and trust in the neighborhood context is also related to parents’ ERS messages and youth’s self-system (Im & Witherspoon, 2025). Neighborhood cohesion and trust represents a type of social capital that increases youth’s access to support and opportunities (e.g., interpersonal, professional, academic) when developing critical attributes such as ERI (Johnston & Soroka, 2003; Spencer, 1995). Though limited work exists on the explicit effects of neighborhood cohesion and trust for the cultural practices of Black and Latino families, Latino parents engaged in more cultural socialization, such as attending traditional celebrations, when they perceived high levels of cohesion (Pasco et al., 2021a). However, for Black parents, scholars have found no significant relationship between cohesion and ERS (Saleem et al., 2016). Neighborhood problems, which are theorized to be negatively associated with cohesion and trust (Shaw & McKay, 1942), are also related to ERS. Witherspoon et al. (2022) found that Black and Latino parents provided more cultural socialization and preparation for bias messages when they perceived high levels of problems in their neighborhood. Additionally, Latino families in new immigrant destination areas, such as the one in the current study, have low ethnic-racial match with their neighbors. Thus, these neighborhoods are known to vary in how welcoming and supportive they are (Flippen & Parrado, 2015). The current study aims to fill this gap in explicit knowledge on neighborhood cohesion and trust’s relation with ERS for these ethnic-racial groups.

A lingering question is whether the neighborhood context has indirect effects on youth’s self-systems through their parents’ ERS practices. Winkler (2012) found that Black mothers in Detroit, a city where Black culture holds a prominent place, relied on their youth’s environment as a primary socializer, and parental ERS messages were mainly given in response to racially charged events. These findings suggest neighborhood context has direct and indirect effects on the developmental outcomes of youth. In their review (31% Latino and 54% Black adolescents), Pasco et al. (2021b) found that neighborhood ethnic-racial affirmation, or living in a neighborhood where one is exposed to the same ethnic-racial group, was positively associated with exploration of youth’s ERI through increased cultural association. Similarly, neighborhood social and cultural cohesion were positively associated with Mexican adolescents’ ERI resolution five years later through increased maternal cultural socialization (Pasco et al., 2021a). Contrastingly, Witherspoon et al. (2021) did not find cultural socialization and preparation for bias beliefs or practices to mediate the relationship between neighborhood diversity and youth ERI in Latino families. The current study aims to fill the gap in knowledge on the mediation effect of egalitarian and promotion of mistrust messages, also expanding the measures of the self-system to include self-esteem. We also look for potential differences in how contextual factors impact self-system development across Latino and Black families to better understand the varying experiences of minoritized youth and families in the United States.

### 1.5. Parental Cultural Stressors and Assets, ERS, and Youth Self-System

Parental cultural experiences play a critical role in shaping ethnic-racial socialization (ERS; García Coll et al., 1996). Two central dimensions of these experiences are cultural stressors and cultural assets. In this study, we focus specifically on perceived discrimination as a cultural stressor and parental ethnic-racial identity (ERI) as a cultural asset. The following sections explore how these two parental cultural factors are theorized to influence youth self-esteem and ERI through their impact on ERS.

#### 1.5.1. Cultural Stressors

Integrative theories posit that Black and Latino parents’ experiences with ethnic-racial discrimination have significant impacts on the messages they present to their children about race and ethnicity (García Coll et al., 1996). Ethnic-racial discrimination is a cultural stressor that is positively associated with ERS messages from Black and Latino parents (Ayón et al., 2020; D. Hughes, 2003; Witherspoon et al., 2022), particularly for preparation for bias and cultural socialization messages with Black families. However, these relations vary by environmental context and type of discrimination experienced (e.g., institutional, interpersonal; Hagelskamp & Hughes, 2014; Holloway & Varner, 2021). For example, Scott and Varner (2023) found that Black parents’ perceived discrimination increased preparation for bias and cultural socialization messages when they worked with few other Black individuals. For Latino parents, unique stressors such as being questioned about their immigrant status, a microaggression, impacted ERS strategies; undocumented Latino parents engaged in more cultural socialization and promotion of mistrust practices with their youth than documented Latino parents (Cross et al., 2020). Further, the neighborhood context, in concert with parents’ discrimination experiences, shaped Black and Latino adolescents’ outcomes (Witherspoon et al., 2022). For example, in low neighborhood cohesion contexts, experiencing more parental discrimination increased promotion of mistrust messages (Saleem et al., 2016).

Parents’ experiences of ethnic-racial discrimination also impact their youth’s self-system. Espinoza et al. (2016) found that Mexican and Mexican American parents’ experiences of discrimination reduced their adolescent’s self-esteem, especially in the context of more frequent ERS messages (cultural socialization, preparation for bias, and promotion of mistrust). Outside of the context of parental discrimination, cultural socialization remained a positive indicator of these youth’s self-esteems. Thus, the authors posit that parents are less likely to relay self-esteem building messages when discussing race and racism with their child as they deal with the stress of experiencing ethnic-racial discrimination. This may be due to the parent’s self-esteem being lowered by the experience of discrimination. To better understand the impacts of parental discrimination on Latino and Black adolescents, the current study explores the mediational effect of ERS beliefs and practices in the association between parental ethnic-racial discrimination and youth self-esteem and ERI for Black and Latino families.

#### 1.5.2. Cultural Assets

Adolescents’ ERI development is intricately influenced by the identity beliefs of their family (Branje et al., 2021). Minoritized parents’ own ERI improves perceptions of their ERS strategies and ability to promote positive well-being in their youth (Kiang et al., 2023). With higher ERI, Black and Latino parents may be better able to self-regulate following discrimination experiences. Theoretically, parents with a strong ERI act as critical role models and a source of support for minoritized adolescents developing their own coping strategies and identities (Spencer, 1995). These positive effects often trickle down to youth’s outcomes (i.e., self-esteem and ERI) directly and through the parents’ ERS beliefs and practices (Derlan et al., 2016; Umaña-Taylor & Hill, 2020). Latino parents with a strong ERI provide their adolescents with more cultural socialization messages (D. Hughes, 2003). Additionally, ERI beliefs in Black mothers are shaped by their past ERS experiences and are positively associated with their ERS messaging to their youth (White-Johnson et al., 2010). The current study examines parents’ ethnic-racial discrimination experiences alongside their ERI, allowing us to see if the two parental characteristics interact such that higher parental ERI in the presence of high parental ethnic-racial discrimination positively associates with youth self-esteem and ERI through ERS messages.

### 1.6. Ethnic-Racial Group as a Moderator

The association between neighborhood, ERS, and youth’s self-system varies by ethnicity. Witherspoon et al. (2022) found that Latino parents engaged in less cultural socialization and preparation for bias messages than Black parents. White et al. (2018) found that Latino parents employed more cultural socialization messaging when in neighborhoods with fewer co-ethnics, or neighbors of the same ethnicity. Barr and Neville (2014) showed that Black parents used more cultural socialization messages when there were more co-ethnics. Yet still, Winkler (2012) showed that Black mothers engaged in fewer cultural socialization messages in a predominantly Black city. Furthermore, cultural socialization and preparation for bias messages have historically been salient in Black families, whereas Latino families’ ERS messages, including egalitarianism, tend to vary by generation and immigrant status (Simon, 2021). To understand the nuances of these ethnic-racial differences in ERS messaging and how these messages associate with the self-system, we explore our models based on ethnic-racial group.

### 1.7. Current Study

**Figure 1:**
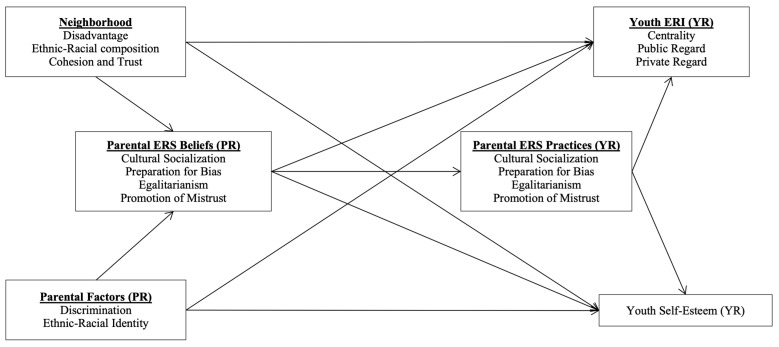
Conceptual model. PR = Parent report. YR = Youth report. ERI = Ethnic-racial identity. ERS = Ethnic-racial socialization. Separate models were run for each outcome. For simplicity, the associations between covariates are also not depicted.

The current study builds on recent empirical research exploring the relationships between neighborhood context, parental cultural factors, and parental ERS and their effects on Latino youth’s ERI in a new destination context (Witherspoon et al., 2021). The present study extends this work by incorporating a more diverse sample and examining broader developmental outcomes. Specifically, it investigates how neighborhood and parental cultural factors shape parental ERS beliefs and practices and adolescent’s self-system (Self-Esteem and ERI) among Black and Latino families residing in a new immigrant destination context. Guided by an ecological framework, this study had three primary aims: (1) to examine how neighborhood (i.e., disadvantage, diversity, and cohesion) and parents’ cultural (i.e., discrimination experiences and ERI) contexts are associated with adolescents’ self-esteem and ERI both directly and indirectly through their influence on parents’ ERS beliefs and practices, (2) to explore whether parents’ ERS beliefs and practices serve as the mediators linking neighborhood and parental cultural contexts and adolescents’ self-esteem and ethnic-racial identity, and (3) explore whether these associations differ between Black and Latino families. The conceptual model is presented in Figure 1.

Grounded in the integrative model (García Coll et al., 1996) and empirical research (White et al., 2018; Witherspoon et al., 2022), we hypothesized that neighborhood stressors (i.e., disadvantage) would be negatively associated with parents’ ERS beliefs, and neighborhood-promotive factors (i.e., diversity, cohesion) would be positively associated with ERS beliefs, such that in higher SES, diverse and cohesive neighborhoods, parents would place greater value on conveying ethnic-racial messages to their children. Also, based on extant scholarship (Holloway & Varner, 2021; D. Hughes et al., 2006), we hypothesized that parents’ cultural factors would be positively associated with more ERS beliefs such that greater discrimination experiences as well as having a stronger ERI would elicit greater belief in the importance of communicating ethnic-racial messages. In line with recent research, we hypothesized that these beliefs would be related positively to youths’ perceptions of ERS practices (Witherspoon et al., 2021). Furthermore, following an accumulation of evidence (Umaña-Taylor & Hill, 2020), we hypothesized that adolescents’ perceptions of cultural socialization practices would be positively associated with all aspects of self-system (i.e., self-esteem, centrality, private regard, and public regard). In contrast, perceptions of preparation for bias socialization were expected to relate negatively to public regard. Furthermore, youth perceptions of egalitarianism practices were hypothesized to be positively associated with self-esteem and private regard, whereas perceptions of promotion of mistrust practices were hypothesized to be negatively associated with self-esteem, centrality, and private and public regard. Additionally, we proposed that parental ethnic-racial socialization beliefs and behaviors would function as mediators linking neighborhood characteristics and parental cultural experiences to adolescent self-esteem and ethnic-racial identity. Last, we hypothesized that there would be ethnic-racial differences in the model overall. Given the exploratory nature of this analysis, we did not have specific hypotheses regarding differential patterns across the associations.

## 2. Methods

### 2.1. Data and Sample

The data for this study came from the Families, Adolescents, and Neighborhoods in Context (FAN-C) Study; a mixed-methods, multi-informant study of ethnic-racially minoritized families in the northeastern United States. The goal of FAN-C was to examine the contribution of neighborhood and cultural factors to parenting practices and youth development. This study was conducted in two phases. Phase I focused on ethnic-racially marginalized families from five distinct neighborhoods within an urban city in the Northeastern urban area, while Phase II only included Latino families who resided anywhere in the city. For the present study, only Black and Latino families, based on caregivers’ self-identification, across both phases are included. Multiple adolescents within a family were allowed to participate in this study, resulting in 144 caregivers and 184 adolescents who participated. Demographic information for the full sample as well as for each ethnic-racial group appears in Table 1. Caregivers^1^ (N = 144) were primarily biological mothers (74.8%) and biological fathers (7.4%), and the remainder were adoptive, step, foster, or grandparents. Parents had an average age of 40.6 years old (SD = 9.16) and identified as Latino/Hispanic (53.5%) or Black/African American (46.5%). Almost one-third (32.6%) were married or cohabitating with a partner. Educational attainment and annual family income were provided to ascertain socioeconomic status. Regarding educational attainment, 16.3% of caregivers did not complete high school; 40.7% obtained a high school degree, 31.1% completed at least some college (including vocational training) and 11.9% completed a bachelor’s degree or higher. The mean annual household income was USD 26,607 (SD = USD 20,948; range = USD 5000–USD 105,000), and the income-to-needs ratio averaged 0.97 (SD = 1.04; range = 0.11–5.28). Only 17.5% of caregivers owned their place of residence. Adolescents (N = 184) ranged in age from 11–17 years old (M = 13.4, SD = 1.91) and were 57.5% female. They were on average in the 8th grade (M = 7.95; SD = 2.14). Youth reported their race as Latino/Hispanic (50.4%), Black/African American (46.8%), and Multiracial (5.5%). Families resided in 22 residential census tracts. Parents resided in their current neighborhood for an average of 8.78 years (SD = 8.71).

### 2.2. Procedure

Participants were recruited through community organizations and events serving Black and Latino families with adolescents aged 11–17 years. The study was advertised at community events, and flyers were placed at locations recommended by community partners. Parents expressed interest in person or via the project hotline. The project coordinator provided more information about the project and received more information about the participant to screen them for eligibility. Eligible families interested in participating in the study were assigned to a focus group session. Focus group sessions began with parents providing informed consent and permission for their youth to participate, and adolescents providing assent. Parents and adolescents completed a questionnaire packet assessing academic achievement, neighborhood perceptions, parental ERS practices, perceived discrimination, and ERI. Parents and adolescents completed the questionnaire packets in separate rooms to ensure confidentiality and to allow adolescents the right to refuse participation at any time. Caregivers chose to complete the survey in either English or Spanish; adolescents completed the surveys in English. Although focus groups were conducted, only questionnaire data were used for the study.

### 2.3. Measures

#### 2.3.1. Demographics

Parents and adolescents reported demographic information. Parents provided demographic information including their relationship to the child, date of birth, race/ethnic background, marital status, education level, annual family income for the previous year, and length of time in their current neighborhood. Youth provided their gender, date of birth, race/ethnic background, and grade. The ages of both parents and youth were computed based on reported birthdates. The income-to-needs ratio (INR) was derived by dividing the family’s reported annual income by the federal poverty threshold relevant to their household size.

#### 2.3.2. Neighborhood Characteristics

Participants’ home addresses were geocoded to determine the census tracts in which they lived (N = 22). Neighborhood economic and racial composition data were retrieved from the 2010 U.S. Census to calculate neighborhood disadvantage and neighborhood ethnic-racial diversity.

Neighborhood disadvantage. Neighborhood disadvantage was assessed using a composite index derived from five census tract-level indicators reflecting economic disadvantage according to social disorganization theory (Shaw & McKay, 1942). The indicators included percentage of female-headed families; percent of unemployed residents 16 and older in the work force; percentage of residents over 25 without a high school degree; percent of residents below poverty level; and residential mobility in the past year. Each indicator was standardized and averaged into a single composite score, where higher values indicate greater levels of neighborhood disadvantage (range = −2.03–1.09). The scale displayed good reliability (α = 0.83).

Ethnic-racial diversity. Neighborhood ethnic-racial diversity was assessed using Simpson’s Index of Diversity (Juvonen et al., 2006; Simpson, 1949) based on 2010 U.S. Census tract racial composition data. The index accounts for both the total number of different ethnic-racial groups preset and the relative distribution of those groups within each neighborhood. The ethnic categories included in the calculation were White, Black/African American, Hispanic/Latino, American Indian or Alaska Native, Asian, Native Hawaiian or Other Pacific Islander, and Other (i.e., some other race not listed or two or more races). Higher values represent greater neighborhood diversity (0–1; range = 0.27–0.67).

Participants lived across a range of neighborhood contexts characterized by varying levels of ethnic-racial composition and diversity. Neighborhoods included those with predominantly White non-Hispanic residents, primarily Black populations, mixed racial-ethnic compositions, and areas experiencing growth in Latino populations. On average, neighborhoods were composed of 53% Black residents (range: 5–84%), 21% White residents (range: 6–78%), 19% Latino residents (range: 0–30%), and approximately 7% representing other racial/ethnic groups, including Asian, American Indian, and Native Hawaiian populations.

Cohesion and trust. Neighborhood cohesion and trust was assessed using an adapted version of the Collective Efficacy Scale (Sampson et al., 1997) designed to measure parents’ perceptions of social connectedness and mutual trust within their neighborhood. The scale consisted of 3 items (e.g., “People in your neighborhood are close”). Parents indicated on a 4-point scale (1 = *completely disagree* to 4 = *agree a lot*) the level of perceived social support present within the neighborhood. The measure displayed good reliability (α = 0.85; Latino α = 0.92, Black α = 0.67).

#### 2.3.3. Parental Cultural Factors^2^

Parental racial discrimination. Parental perceived racial discrimination was assessed using an adapted version of the Everyday Discrimination Scale (D. R. Williams et al., 1997) expanded to include items specific to experiences at their children’ school (Seaton et al., 2008). The 13-item scale (e.g., How often are you treated with less courtesy than other people because you are [ethnic group]?) measures parents’ perceptions of how often they experienced unfair treatment. Parents rated items on a 5-point scale (1 = *never* to 5 = *very often*). Higher scores indicated more frequent racial discrimination. The measure demonstrated good reliability (α = 0.95, Latino α = 0.96, Black α = 0.95).

Parental ethnic-racial identity. Parents’ ERI was assessed with The Multi-Group Ethnic Identity Measure (MEIM; Phinney, 1992), a 12-item scale that measures two aspects of ERI. The exploration subscale measures the extent to which individuals actively seek to learn about their ethnic-racial background heritage (e.g., I have a clear sense of my ethnic background and what it means for me), and the affirmation and belonging subscale measures individuals’ feelings of pride and attachment to their ethnic group (e.g., I have a lot of pride in my ethnic group). Parents rated each item on a 4-point scale (1 = *strongly agree* to 4 = *strongly disagree*). All items were averaged to create a total ERI score, with higher scores reflecting a stronger ethnic-racial identity. The measure demonstrated good reliability (α = 0.91, Latino α = 0.92, Black α = 0.91).

#### 2.3.4. Ethnic-Racial Socialization

Parental ethnic-racial socialization beliefs—parent report. Parents’ beliefs about ethnic-racial socialization (ERS) were assessed using the ethnic-racial socialization measure (D. Hughes et al., 2008). This measure assessed parents’ attitudes about the importance of four types of ERS: cultural socialization (e.g., make sure their children maintain [ethnic group] values and beliefs), *preparation for bias* (e.g., prepare [ethnic group] children to cope with discrimination), egalitarianism (e.g., encourage their children to have friends of all races and ethnicities?), and promotion of mistrust (e.g., teach children not to trust people who are not [ethnic group]?). The cultural socialization, preparation for bias, and egalitarianism subscales each included four items, while the promotion of mistrust subscale included two items. Parents rated items on a 4-point scale (1 = *not at all important* to 4 = *very important*), with higher scores indicating a stronger ERS belief. The four scales demonstrated good reliability overall (α_cultural socialization_ = 0.72, α_preparation for bias_ = 0.78, α_egalitarianism_ = 0.78, Spearman–Brown_promotion of mistrust_ = 0.71 and within each ethnic-racial group (Latino: α_cultural socialization_ = 0.81, α_preparation for bias_ = 0.80, α_egalitarianism_ = 0.81, Spearman–Brown_promotion of mistrust_ = 0.69; Black α_cultural socialization_ = 0.66, α_preparation for bias_ = 0.71, α_egalitarianism_ = 0.69, Spearman–Brown_promotion of mistrust_ = 0.60). The cultural socialization subscale was highly correlated with the preparation for bias (*r* = 0.71, *p* < 0.001) and egalitarianism (*r* = 0.80, *p* < 0.001) subscales. To avoid multicollinearity, exploratory factor analysis was conducted to determine whether the constructs could be combined. Across both ethnic-racial groups, one factor was extracted for the cultural socialization and egalitarianism items. The items were averaged to create a cultural-egalitarianism scale that showed good reliability (α = 0.87, Latino: α = 0.91; Black: α = 0.84). The reliability was higher than for the individual subscales.

Parental ethnic-racial socialization practices—adolescent report. The youth version of the ethnic-racial socialization measure (D. Hughes et al., 2009b) assessed adolescents’ perceptions of how often their parents engaged in ERS practices. The measure captured four types of ERS: cultural socialization, preparation for bias, egalitarianism, and promotion of mistrust. Cultural socialization was measured with three items (e.g., How often have your parents said be proud of your race?). Preparation for bias (e.g., How often have your parents said people will treat you unfairly?) and egalitarianism (e.g., How often have your parents said people are all equal, regardless of their race or ethnicity?) were each measured with six items. Promotion of mistrust was assessed with two items (e.g., How often have your parents said things like it is a bad idea to date (or go out with) someone who is a different race or ethnicity than you are?). Adolescents rated each item on a 3-point scale (1 = *never* to 3 *= a lot of times*). The four scales demonstrated good reliability overall (α_cultural socialization_ = 0.77, α_preparation for bias_ = 0.75, α_egalitarianism_ = 0.78, Spearman–Brown_promotion of mistrust_ = 0.91) and within each racial-ethnic group (Latino: α_cultural socialization_ = 0.74, α_preparation for bias_ = 0.77, α_egalitarianism_ = 0.79, Spearman–Brown_promotion of mistrust_ = 0.84; Black: α_cultural socialization_ = 0.78, α_preparation for bias_ = 0.77, α_egalitarianism_ = 0.75, Spearman–Brown_promotion of mistrust_ = 0.95. The cultural socialization and egalitarian subscales were highly correlated, *r* = 0.70, *p* < 0.001. Exploratory factor analysis was conducted to determine if the types could be combined. One factor was extracted, and the cultural socialization and egalitarian items were averaged to create a cultural-egalitarianism scale that showed good reliability (α = 0.85; Latino: α = 0.87; Black: α = 0.81). The reliability was higher than for the individual subscales.

#### 2.3.5. Adolescent Outcomes

Self-esteem. Adolescents’ self-esteem was assessed using the Rosenberg Self-Esteem Scale (Rosenberg, 1979), a 10-item (e.g., I feel that I have a number of good qualities) self-report scale that measures feelings of self-worth and self-acceptance. Adolescents rated on a 4-point scale (0 = *strongly disagree* to 3 = *strongly agree*) how they feel about themselves, with higher scores indicating greater self-esteem. The measure demonstrated good reliability (α = 0.80; Latino: α = 0.74; Black: α = 0.81).

Ethnic-racial identity. Adolescents’ ethnic-racial identity (ERI) was assessed using an adapted version of the Multidimensional Inventory of Black Identity (Sellers et al., 1998). Adolescents’ centrality (4 items; e.g., “I have a strong attachment to my ethnicity”), private regard (4 items; e.g., “I am happy about my ethnicity”), and public regard (4 items; e.g., “A lot of people don’t expect my ethnic group to do well in life”) were each measured with four item subscales. Two negatively worded items in the public regard subscale were reverse-coded so that, across all subscales, higher scores reflected higher levels of each construct. Adolescents rated each item on a 5-point scale (1 = *strongly disagree* to 5 = *strongly agree*). The scales demonstrated acceptable reliability overall (α_centrality_ = 0.70, α_private regard_ = 0.69, α_public regard_ = 0.62) and within each ethnic-racial group (Latino: α_centrality_ = 0.67, α_private regard_ = 0.68, α_public regard_ = 0.58; Black: α_centrality_ = 0.61, α_private regard_ = 0.52, α_public regard_ = 0.71).

### 2.4. Data Analysis Plan

The proposed model was tested using path analysis with full-information maximum likelihood (FIML) estimation to address missing data and 95% bias-corrected bootstrapped confidence intervals to assess the significance of indirect effects. Given the hierarchical nature of the data, families nested within neighborhoods, intraclass correlation coefficients (ICC) were calculated to evaluate the potential impact of clustering at the neighborhood level for all variables except for neighborhood disadvantage and neighborhood diversity, which were constant within neighborhoods. The ICC analyses revealed that most variables showed negligible between-neighborhood variability, with ICCs below 0.1 (ranging from 0.000–0.095). An exception was found for parental promotion of mistrust beliefs, which had an ICC of 0.115, indicating that a small but meaningful proportion of its variance (11.5%) was attributable to neighborhood-level differences. Additionally, the model was also estimated using maximum likelihood with robust standard errors (MLR) to account for the non-independence of observations (Hox et al., 2010). However, because bootstrapping methods (Preacher & Hayes, 2008) cannot be applied simultaneously with MLR, separate analyses were conducted using each approach. The results from both methods were then compared to ensure consistency and robustness. The significance and direction of effects was similar across models, so we chose to report the bootstrapped estimates, given their relevance for testing mediation, which were central to our research questions.

Model fit was assessed using multiple indices, including the chi-square statistic, root mean square error of approximation (RMSEA), comparative fit index (CFI), Tucker–Lewis Index (TLI), and standardized root mean square residual (SRMR). Following conventional guidelines, acceptable model fit was indicated by RMSEA values ≤ 0.06, CFI ≥ 0.95, TLI values ≥ 0.90, and SRMR values ≤ 0.08. Bias-corrected bootstrap confidence intervals of 95% based on 10,000 bootstrap samples (Preacher et al., 2007) were used to test the significance of indirect effects. Covariates that were significantly correlated (*p* < 0.01) with study variables were included as predictors in the relevant paths. All analyses were conducted in Mplus Version 8.11 (Muthén & Muthén, 1998–2017).

Multigroup analysis was conducted to test for ethnic-racial group as a moderator. For these analyses, separate models were estimated for each group. Then, two nested models were tested: one in which all structural paths were allowed to vary across groups (unconstrained), and another in which all paths were constrained to be equal (constrained). A chi-square difference test was used to determine whether model fit significantly improved when allowing parameters to differ by group. Pairwise parameter comparisons were used to identify significant differences (*p* < 0.10) in paths between Black and Latino families. When the chi-square difference test suggested significant group differences, specific paths were systematically freed based on pairwise parameter comparisons. This iterative process continued until freeing additional paths no longer improved model fit, as determined by the chi-square difference test. The final models included constrained and free paths.

## 3. Results

### 3.1. Preliminary Analysis

*T*- and chi-square tests were used to examine differences between Black and Latino families in demographics and study variables. As shown in Table 1, on average, Black parents had higher levels of education, higher income, were more likely to be single (i.e., not married or cohabiting) and less likely to be separated/widowed/divorced, and lived in the neighborhoods longer than Latino parents. Additionally, Black parents lived in less disadvantaged and more cohesive neighborhoods, reported less belief in promotion of mistrust messaging, and their youth reported less perception of parental cultural-egalitarian practices and higher self-esteem. Table 2 contains bivariate correlations among covariates and study variables for each racial-ethnic group.

### 3.2. Structural Equation Modeling

**Figure 2:**
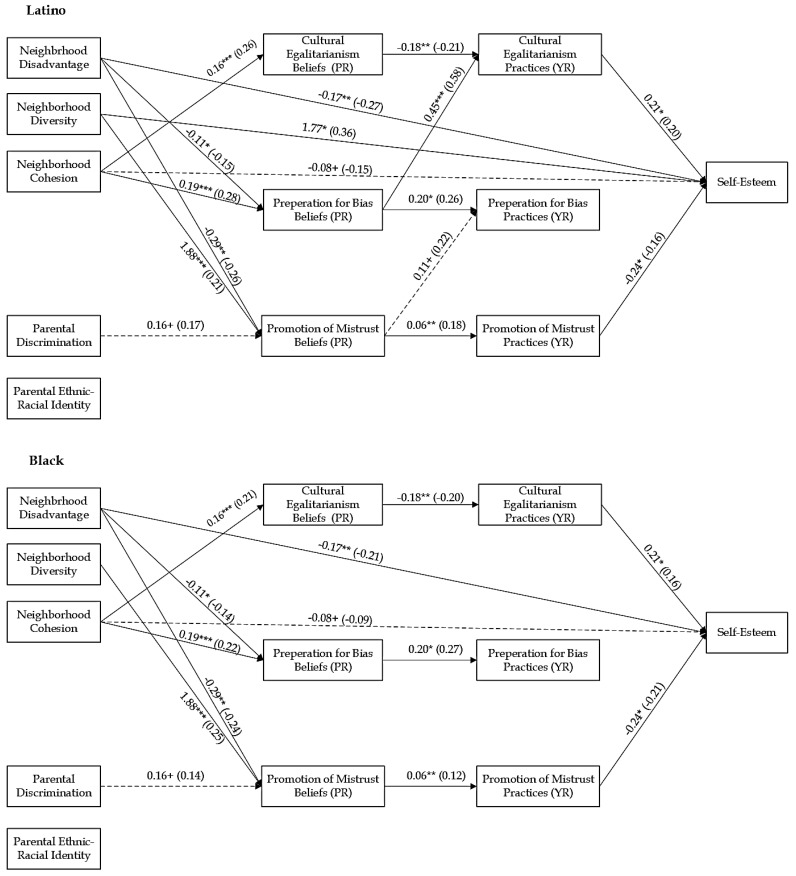
Latino and Black multigroup analysis for self-esteem. Unstandardized results and standardized results (in parentheses) are reported for the significant and marginally significant paths. Solid lines represent significant associations. Dotted lines represented marginally significant associations. + *p* < 0.10, * *p* < 0.05, ** *p* < 0.01, *** *p* < 0.001.

**Figure 3:**
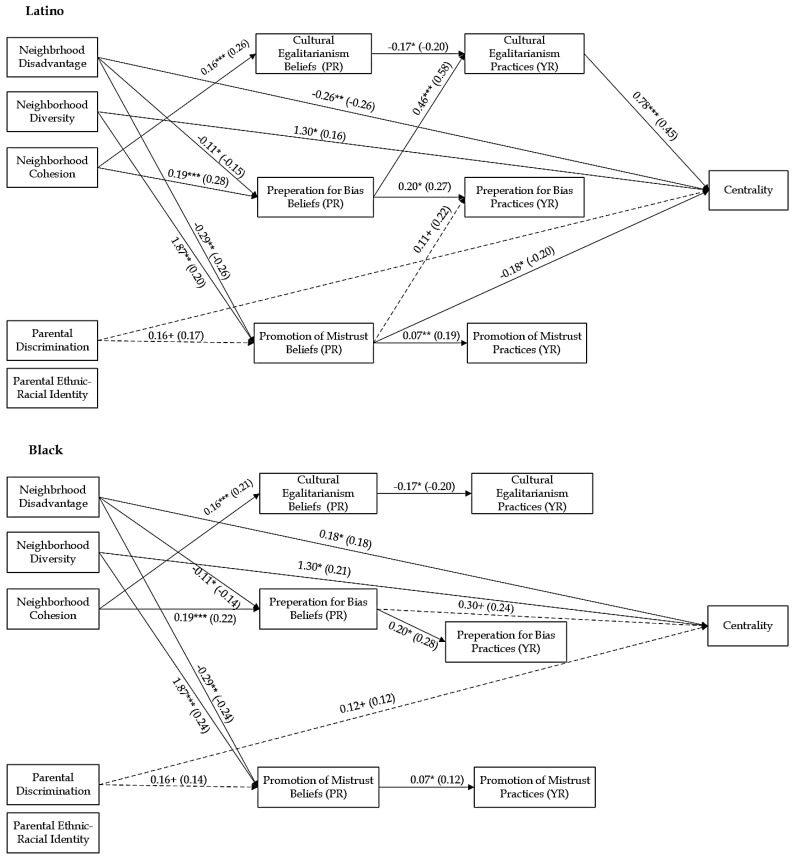
Latino and Black multigroup analysis for centrality. Unstandardized results and standardized results (in parentheses) are reported for the significant and marginally significant paths. Solid lines represent significant associations. Dotted lines represented marginally significant associations. + *p* < 0.10, * *p* < 0.05, ** *p* < 0.01, *** *p* < 0.001.

**Figure 4:**
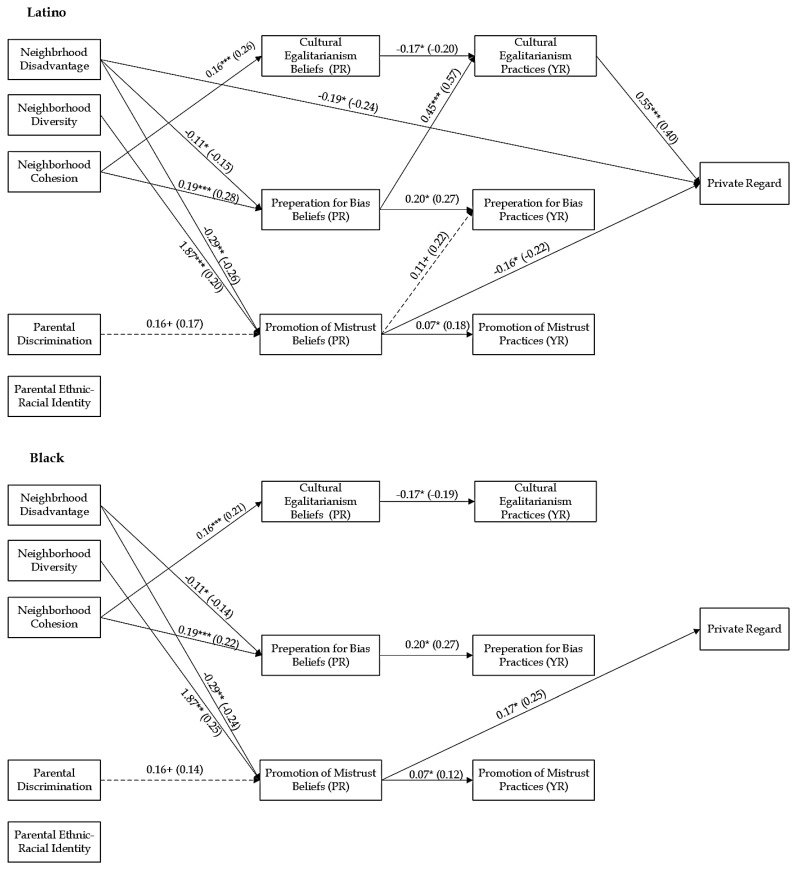
Latino and Black multigroup analysis for private regard. Unstandardized results and standardized results (in parentheses) are reported for the significant and marginally significant paths. Solid lines represent significant associations. Dotted lines represented marginally significant associations. + *p* < 0.10, * *p* < 0.05, ** *p* < 0.01, *** *p* < 0.001.

**Figure 5:**
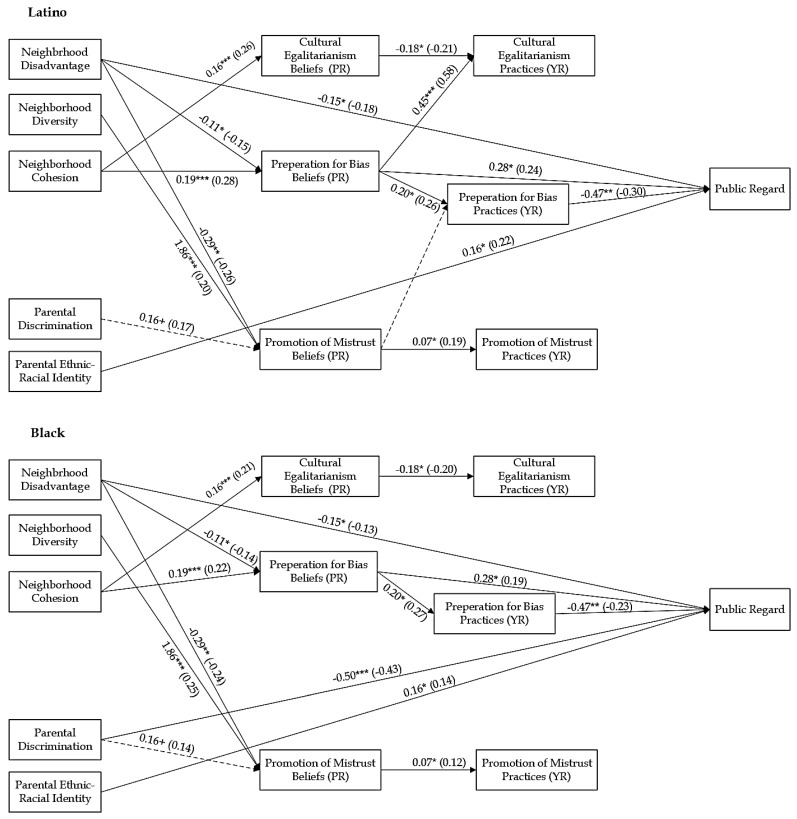
Latino and Black multigroup analysis for public regard. Unstandardized results and standardized results (in parentheses) are reported for the significant and marginally significant paths. Solid lines represent significant associations. Dotted lines represented marginally significant associations. + *p* < 0.10, * *p* < 0.05, ** *p* < 0.01, *** *p* < 0.001.

To test for ethnic-racial group differences, the unconstrained models were compared to fully constrained models. The chi-square difference between the freely estimated and constrained models was significant (Self-Esteem: Δχ^2^ (35) = 54.87, *p* < 0.05; Centrality: Δχ^2^ (35) = 75.74, *p* < 0.001; Private Regard: Δχ^2^ (35) = 74.19, *df* = 35, *p* < 0.001; Public Regard: Δχ^2^ (35) = 62.14, *p* < 0.01), indicating that the regression coefficients of the paths were different for Black and Latino families. Pairwise parameter comparisons were used to examine ethnic-racial differences in pathways (Table S1). The constrained model was used to test the identified paths in the model sequentially to confirm they were significantly different based on ethnic-racial group. In each model, for all the identified pathways, the chi-square differences were significant (all *p*s < 0.05; Table 3). The final partially constrained models (see Figure 2, Figure 3, Figure 4 and Figure 5) demonstrated good fit: Self-Esteem: χ^2^ (35) = 30.43, *p* = 0.69, RMSEA = 0.00, CFI = 1.00, TLI = 1.00, and SRMR = 0.04; Centrality: χ^2^ (35) = 27.91, *p* = 0.80, RMSEA = 0.00, CFI = 1.00, TLI = 1.00, and SRMR = 0.04; Private Regard: χ^2^ (35) = 28.15, *p* = 0.79, RMSEA = 0.00, CFI = 1.00, TLI = 1.00, and SRMR = 0.05; Public Regard: χ^2^ (35) = 31.96, *p* = 0.62, RMSEA = 0.00, CFI = 1.00, TLI = 1.00, and SRMR = 0.04. Table 4 displays the significant indirect effects. We provide results based on each substantive variable, beginning with those parameters that are similar between Black and Latino families, followed by reporting results of those parameter estimates that differed between ethnic-racial groups. All direct and indirect effects for all models are reported in the Supplemental Materials (Table S2).

### 3.3. Direct Effects

#### 3.3.1. Effects of Neighborhood Factors on ERS and Youth Self-System

Neighborhood factors had direct associations with youth’s self-system. For both Latino and Black families, neighborhood disadvantage was associated with lower self-esteem (b = −0.17, *p* < 0.01) and public regard (b = −0.15, *p* < 0.05). There was a significant difference between Black and Latino families in the association between neighborhood disadvantage and youth’s centrality (z = −3.39, *p*= < 0.01) and private regard (z = −3.00, *p* < 0.01). In Latino families, neighborhood disadvantage was associated with less centrality (b = −0.26, *p* < 0.01) and private regard (b = −0.19, *p* < 0.05). However, in Black families, neighborhood disadvantage was associated with greater centrality (b = 0.18, *p* < 0.05) and not significantly associated with youth’s private regard (b = 0.13, *p* = 0.14). Neighborhood diversity was associated with higher levels of centrality (b = 1.30, *p* < 0.05) for all families. There were marginal ethnic-racial differences in the association between neighborhood diversity and self-esteem (z = 1.65, *p* < 0.10). In Latino families, there was a positive association with youth’s self-esteem (b = 1.77, *p* < 0.05) but no association (b = 0.26, [−0.68, 1.31], *p* = 0.60) in Black families. Neighborhood cohesion was not significantly associated with youth’s ERI but was marginally associated with less self-esteem (b = −0.08, *p* < 0.10).

For all, neighborhood factors were associated with parental ERS beliefs. Across all models, neighborhood disadvantage was associated with less belief in the importance of preparation for bias (bs = −0.11, *p*s < 0.05) and promotion of mistrust (bs = −0.29, *p*s < 0.01), whereas neighborhood diversity was associated with greater belief in the importance of the promotion of mistrust (bs = 1.88, *p*s < 0.01). Neighborhood cohesion was associated with greater parental belief in the importance of cultural-egalitarianism (bs = 0.16, *p*s < 0.001) and preparation for bias (bs = 0.19, *p*s < 0.001).

#### 3.3.2. Effects of Parental Cultural Factors on ERS and Youth Self-System

Parental discrimination was marginally associated with higher levels of centrality for all youth (b = 0.12, *p* < 0.10). There were significant differences between Latino and Black families in the association between parental discrimination and public regard (z = 3.26, *p* < 0.01). In Black families, parental discrimination was associated with less public regard (b = −0.50, *p* < 0.001); there was no relation for Latino families (b = −0.02, *p* = 0.80). Furthermore, parental ERI was related to greater public regard (b = 0.16, *p* < 0.05) for all youth. Additionally, parental discrimination was marginally associated with greater belief in the importance of promotion of mistrust (bs = 0.16, *p*s < 0.10) for all families.

#### 3.3.3. Effects of ERS Beliefs on ERS Practices and Youth Self-System

Parents’ ERS beliefs were associated with youth’s outcomes and perceptions of parental ERS practices. Parents’ preparation for bias beliefs was associated with higher levels of public regard (b = 0.28, *p* < 0.05). There were significant ethnic-racial differences in the association of parents’ preparation for bias beliefs and youth’s centrality (z = −2.12, *p* < 0.05). In Black families, parents’ preparation for bias beliefs was marginally associated with higher levels of centrality (b = 0.30 [−0.04, 0.65], *p* < 0.10), while there was no significant association for youth in Latino families (b = −0.15 [−0.48, 0.20], *p* = 0.40). There were also ethnic-racial differences in the associations between parental promotion of mistrust beliefs and youth’s centrality (z = −2.14, *p* = 0.03) and private regard (z = −3.48, *p* < 0.01). In Latino families, promotion of mistrust beliefs was associated with lower levels of centrality (b = −0.18 [−0.34, −0.01], *p* < 0.05) and private regard (b = −0.16 [−0.30, −0.02], *p* < 0.05), while in Black families, it was not associated with youth’s centrality (b = 0.07, *p* = 0.37) and was associated with higher levels of private regard (b = 0.17, *p* < 0.05).

In terms of youth’s perception of ERS practices, for all families, parents’ cultural-egalitarianism beliefs were associated with less perceived cultural-egalitarianism (bs = −0.17, −0.18, *p*s < 0.05), while parental preparation for bias beliefs was associated with greater perceived preparation for bias (bs = 0.20, *p*s < 0.05), and parental promotion of mistrust beliefs was associated with greater perceived promotion of mistrust (bs = 0.06, *p*s < 0.05). There were ethnic-racial differences in the association between parental preparation for bias beliefs and youth’s perceptions of parental cultural-egalitarianism practices (zs = 4.22, *p*s < 0.001), as well as the promotion of mistrust beliefs and perceived preparation for bias practices (zs = −2.42, *p*s = 0.02). In Latino families, parental preparation for bias beliefs was associated with more youth perceived cultural-egalitarianism (bs = 0.45 [0.27, 0.63], *p*s < 0.001), and parental promotion of mistrust beliefs was marginally associated with greater perceived promotion of mistrust (bs = 0.11, *p*s < 0.10), but these associations were not significant in Black families (bs = −0.04], *p*s = 0.70 and bs = −0.09 [−0.21, 0.05], *p*s = 0.16, respectively).

#### 3.3.4. Effects of ERS Practices on Youth Self-System

Youth’s perceptions of parental ERS were associated with their self-esteem and ERI. Youth’s perceptions of cultural-egalitarianism were associated with higher self-esteem (b = 0.21, *p* < 0.05), while their perception of promotion of mistrust was associated with less self-esteem (b = −0.24 [−0.46, −0.02], *p* < 0.05). Youth’s perception of preparation for bias was associated with lower levels of public regard (b = −0.47 [−0.73, −0.18], *p* < 0.01). There were significant ethnic-racial differences in the association of youth’s perceptions of cultural-egalitarianism and youth’s centrality (z = 3.35, *p* < 0.01) and private regard (z = −2.79, *p* < 0.01). In Latino families, youth’s perceptions of cultural-egalitarianism were associated with higher centrality (b = 0.78, *p* < 0.001) and private regard (b = 0.55, *p* < 0.001), while there were no significant associations in Black families (b = −0.24, *p* = 0.34 and b = −0.25 [−0.64, 0.18], respectively).

### 3.4. Mediation Effects

#### 3.4.1. Self-Esteem

Regarding the mediation effects, neighborhood and parental factors had indirect effects on youth’s self-esteem. For all families, neighborhood disadvantage, diversity, and cohesion showed significant indirect effects. Neighborhood disadvantage had a positive indirect effect on youth’s self-esteem sequentially through parents’ promotion of mistrust beliefs and practices, b = 0.004, [0.001, 0.02]. Neighborhood diversity had a significant negative indirect effect sequentially through parents’ belief in the promotion of mistrust beliefs and practices, b = −0.01, [−0.02, −0.001]. Last, neighborhood cohesion had a negative indirect effect through parents’ cultural-egalitarianism beliefs and practices b = −0.01, [−0.02, −0.001]. Additionally, there were significant indirect effects for Latino families only. Neighborhood disadvantage had a negative indirect effect on youth’s self-esteem sequentially through parents’ preparation for bias beliefs and youth’s perception of cultural-egalitarianism, b = −0.01, [−0.04, −0.001]. Neighborhood diversity had a marginal negative total indirect effect, b = −0.17, [−0.046, 0.03], resulting from a marginally negative indirect effect sequentially through promotion of mistrust beliefs and youth’s perception of preparation for bias practices, b = −0.03, [−0.10, 0.002]. Last, neighborhood cohesion had a positive total indirect effect on youth’s self-esteem, b = 0.03 [0.002, 0.08], due to a positive indirect effect sequentially through parents’ preparation for bias beliefs and youth’s perception of cultural-egalitarianism, b = 0.02, [0.004, 0.05].

In terms of ERS beliefs and self-esteem, for all families, parents’ cultural-egalitarianism beliefs had a negative indirect effect through youth’s perception of their cultural-egalitarianism, b = −0.04, [−0.12, −0.002], parents’ preparation for bias beliefs had a marginal negative indirect effect through youth’s perception of their preparation for bias practices, b = −0.03, [−0.09, −0.003], and parents’ promotion of mistrust beliefs had a negative indirect effect through youth’s perception of their promotion of mistrust practices, b = −0.02, [−0.04, −0.003]. Additionally, in Latino families, preparation for bias beliefs had a positive indirect effect through youth’s perception of their cultural-egalitarianism practices, b = 0.09 [0.01, 0.19], and there was a negative total indirect effect from promotion of mistrust beliefs to self-esteem, b = −0.03 [−0.08, −0.01].

#### 3.4.2. Centrality

As hypothesized, there were ethnic-racial differences in the indirect effects of neighborhood and parental factors on youth’s self-system, specifically for ERI centrality. In Black families, neighborhood disadvantage and cohesion had total indirect effects on youth’s centrality (b_Ndis_ = −0.05, [−0.13, −0.002] and b_Ncoh_ = 0.06 [0.01, 0.13], respectively). Specifically, the indirect effect through parents’ preparation for bias beliefs was negative for neighborhood disadvantage, b = −0.03, [−0.11, −0.002], and positive for neighborhood cohesion, b = 0.06 [0.001, 0.15]. However, in Latino families, neighborhood disadvantage had a positive indirect effect on youth’s centrality through parents’ promotion of mistrust beliefs, b = 0.05, [0.01, 0.13], and it had a negative indirect effect sequentially through parents’ preparation for bias beliefs and youth’s perception of cultural-egalitarianism, b = −0.04, [−0.12, −0.01]. Neighborhood cohesion had a positive indirect effect sequentially through parents’ preparation for bias beliefs and youth’s perception of cultural-egalitarianism b = 0.07, [0.03, 0.16], as well as a negative indirect effect sequentially through parents’ cultural-egalitarianism beliefs and youth’s perception of cultural-egalitarianism practices b = −0.02, [−0.07, −0.004]. Additionally, neighborhood diversity had a significant negative total indirect effect on Latino youth’s centrality, b = −0.46, [−1.02, −0.01], due to a negative indirect effect through parents’ belief in the promotion of mistrust, b =−0.33 [−0.80, −0.04]. Parental discrimination had a marginal negative indirect effect through parents’ promotion of mistrust beliefs and practices, b = −0.03, [−0.09, 0.00], and parental ERI had a marginal negative indirect effect sequentially through parents’ cultural-egalitarianism beliefs and practices, b = −0.01 [−0.04, 0.00].

#### 3.4.3. Private Regard

There were also ethnic-racial differences in the indirect effects of neighborhood and parental factors on private regard. In Black families, neighborhood disadvantage had a total indirect effect on youth’s private regard, b = −0.06, [−0.13, −0.002], due to a negative indirect effect through parents’ promotion of mistrust beliefs, b = −0.05, [−0.12, −0.01], and neighborhood diversity had a significant total positive indirect effect on youth’s private regard, b = 0.30, [0.03, 0.69]. Specifically, there was a significant positive indirect effect through parents’ belief in the promotion of mistrust beliefs, b = 0.31, [0.07, 0.72]. Parental discrimination had a positive indirect effect on youth’s private regard through parents’ promotion of mistrust beliefs, b = 0.03, [0.001, 0.08]. However, in Latino families, neighborhood disadvantage had a positive indirect effect through parents’ promotion of mistrust beliefs, b = 0.05, [0.01, 0.11], and it had a negative indirect effect sequentially through parents’ preparation for bias beliefs and youth’s perception of cultural-egalitarianism, b = −0.03, [−0.08, −0.01]. Additionally, neighborhood diversity had a significant negative total indirect effect on youth’s private regard, b = −0.38, [−0.85, −0.09], resulting from a negative indirect effect through parents’ belief in the promotion of mistrust beliefs, b = −0.30, [−0.69, −0.07]. Neighborhood cohesion had an indirect effect sequentially through parents’ belief in preparation for bias and youth’s perceptions of cultural-egalitarianism b = 0.05, [0.02, 0.11], as well as through parents’ cultural-egalitarianism beliefs and practices, b = −0.02, [−0.04, −0.003]. Parental discrimination had a negative indirect effect through parents’ promotion of mistrust beliefs, b = −0.03, [−0.08, −0.001], and parental ERI had a marginal negative indirect effect through cultural-egalitarianism belief and practices b = −0.01, [−0.03, 0.00].

In terms of ERS beliefs and youth’s centrality and private regard, there were only significant indirect effects in Latino families. For both outcomes, parents’ cultural-egalitarianism beliefs had a marginal negative total indirect effect (b = −0.14, [−0.33, 0.00] and b = −0.09, [−0.22, 0.01], respectively). Specifically, there were negative indirect effects through youth’s perception of parents’ cultural-egalitarianism practices (b = −0.14, [−0.33, 0.00] and b = −0.09, [−0.22, −0.01], respectively). Additionally, parents’ preparation for bias beliefs had positive total indirect effects on youth’s centrality (b = 0.38 [0.19, 0.67]) and private regard (b = 0.26 [.13, 0.45]). Specifically, parents’ preparation for bias beliefs had positive indirect effects through youth’s perception of their parents’ cultural-egalitarianism practices (b = 0.36, [0.17, −0.65] and b = 0.25 [0.12, 0.44], respectively).

#### 3.4.4. Public Regard

For all families, neighborhood disadvantage had significant and marginal indirect effects on youth’s public regard through parents’ preparation for bias beliefs (b = −0.03, [−0.10, −0.004]), promotion of mistrust beliefs (b = −0.03, [−0.10, −0.004]), and sequentially through parents’ promotion of mistrust beliefs and preparation for bias practices (b = 0.01, [0.002, 0.04]). In Black families, this resulted in a significant negative total indirect effect of neighborhood disadvantage, b = −0.05 [−0.12, 0.01]. In Latino families, there was a significant indirect effect of neighborhood disadvantage through parents’ promotion of mistrust beliefs and youth’s perceptions of preparation for bias, b = 0.01, [0.001, 0.04], and there was a marginal negative indirect effect through parents’ preparation for bias beliefs and youth’s perception of cultural-egalitarianism, b = −0.01, [−0.04, 0.00]. Neighborhood diversity had indirect effects marginally through promotion of mistrust beliefs, b = 0.20 [−0.03, 0.61], and in Latino families only, sequentially through parents’ promotion of mistrust beliefs and youth’s perception of preparation for bias, b = −0.09, [−0.28, −0.01]. Neighborhood cohesion had significant and marginal indirect effects on youth’s public regard through parents’ preparation for bias, b = 0.05, [0.01, 0.14], and cultural-egalitarianism (b = −0.04, [−0.10, 0.01]) beliefs as well as significantly sequentially through parents’ preparation for bias beliefs and practices (b = −0.02, [−0.06, −0.003]), and marginally sequentially through parents’ cultural-egalitarianism beliefs and youth’s perception of preparation for bias, b = 0.02, [−0.002, 0.06]. Additionally, in Latino families only, there was a marginal indirect effect sequentially through parents’ preparation for bias beliefs and youth’s perception of cultural-egalitarianism, b = 0.02, [−0.002, 0.07].

In terms of ERS beliefs and youth’s public regard, parents’ cultural-egalitarianism beliefs had a marginal indirect effect through youth’s perception of their preparation for bias, b = 0.08, [−0.01, 0.23], and parents’ preparation for bias beliefs had an indirect effect through youth’s perception of their preparation for bias practices, b = −0.09, [−0.24, −0.01]. Additionally, in Latino families, parents’ promotion of mistrust beliefs had a negative total indirect effect on youth’s public regard, b = −0.06 [−0.14, −0.002].

#### 3.4.5. Summary

As summarized in Table 4, three models of indirect effects emerged—one each for self-esteem, ERI positive affect (centrality and private regard), and public regard. Most self-esteem-related indirect effects were found in Latino families. For Black families, only the effects common across both groups were observed: both positive and negative indirect effects of neighborhood disadvantage and diversity via parents’ promotion of mistrust, and a negative effect of neighborhood cohesion via cultural egalitarianism. In Latino families, additional effects included a negative indirect effect through parents’ preparation for bias and youth’s perception of cultural-egalitarianism; a marginal negative indirect effect of neighborhood diversity via promotion of mistrust and preparation for bias; and a positive indirect effect of neighborhood cohesion via preparation for bias and cultural-egalitarianism, leading to a positive total effect. For centrality and private regard, findings varied by ethnic-racial group, with more indirect effects in Latino than Black families. In Latino families, neighborhood disadvantage had both positive (via promotion of mistrust beliefs) and negative (via preparation for bias beliefs and cultural-egalitarianism practices) indirect effects. In contrast, in Black families, neighborhood disadvantage only had one indirect effect, a negative indirect effect on centrality (via preparation for bias beliefs) and on private regard (via preparation for bias beliefs and cultural-egalitarianism practices). Overall, this resulted in negative total indirect effects of neighborhood disadvantage on centrality and private regard in Black families.

Neighborhood diversity through promotion of mistrust beliefs had negative effects in Latino families but nonsignificant (centrality) or positive (private regard) effects in Black families, resulting in negative total indirect effects in Latino families and a positive total indirect effect on private regard in Black families. In Latino families only, neighborhood cohesion had both negative (via cultural egalitarianism) and positive (via preparation for bias and youth perceptions) indirect effects. In Black families, there was only a positive indirect effect through preparation for bias beliefs, resulting in a positive total indirect effect. Lastly, parental discrimination had opposing effects on private regard through promotion of mistrust—negative in Latino families and positive in Black families.

For public regard, the indirect effects were similar across ethnic-racial groups. For all families, neighborhood disadvantage had negative indirect effects through preparation for bias beliefs and promotion of mistrust beliefs marginally and a positive indirect effect sequentially through preparation for bias beliefs and practices. Neighborhood diversity had a marginally positive indirect effect through promotion of mistrust beliefs. Neighborhood cohesion had both marginally negative (via cultural-egalitarianism beliefs and sequentially through preparation for bias beliefs and practices) and positive indirect effects (via preparation for bias beliefs, and sequentially through cultural-egalitarianism beliefs and preparation for bias practices). In Latino families only, neighborhood disadvantage had a marginal negative and a positive indirect effect sequentially, through preparation for bias beliefs and cultural-egalitarianism practices and promotion of mistrust beliefs and preparation for bias practices; neighborhood diversity had an additional indirect effect, sequentially promoting mistrust beliefs and preparation for bias practices; and neighborhood cohesion had a marginal positive indirect effect sequentially through preparation for bias beliefs and cultural-egalitarianism practices.

## 4. Discussion

The current study contributes to the growing body of research on ethnic-racial socialization (ERS) by examining its role in the impact of neighborhood and parental cultural contexts on components of adolescent’s self-system (self-esteem and ERI) in Black and Latino families in a new destination context. While prior research has separately explored the role of neighborhood and parental cultural factors on ERS and youth’s self-system, few have considered how these contextual factors simultaneously influence youth’s self-esteem and ERI with ERS as a potential mediating mechanism. This study expands our understandings of how culturally relevant parenting unfold in Black and Latino families in various contexts, highlighting the importance of context in studies of race and ethnicity in the modern U.S. landscape.

### 4.1. ERS Beliefs and Practices and Youth’s Self-System

Parents and adolescents in Black and Latino families did not differentiate between cultural socialization and egalitarianism beliefs and perceived practices. This may be shaped by the broader social and neighborhood contexts in which these families reside. In particular, for Black and Latino families in a new destination context that consisted of Latino families immigrating to mostly Black neighborhoods, there may be a heightened need to balance the transmission of cultural heritage with messages that encourage respect and inclusion across racial and ethnic groups. In such contexts, promoting both cultural pride and intergroup understanding may be seen as equally essential. In line with this idea, prior research has shown that ethnic-racially minoritized families socialize their children through a stronger focus on intergroup relations (Priest et al., 2014). Additionally, in a new destination context, youth may experience racism or xenophobia, and part of the parental response to this can be both reaffirming their cultural heritage and pride (through cultural socialization) and promoting a commitment to equality (through egalitarianism) so their children understand their value while also advocating for fair treatment for all people, regardless of race or ethnicity. This is aligned with Smith-Bynum’s (2023) concept of affirming egalitarianism, which is parental communication found in Black families that affirms the child’s worth and dignity by emphasizing racial pride and messages of equality in response to experiences or concerns related to racial discrimination. Our finding that preparation for bias beliefs was highly correlated with greater cultural-egalitarianism beliefs and was associated with greater perceived cultural-egalitarianism practices for Latino families offers partial support these assumptions.

As hypothesized, parents’ ERS beliefs were associated with youth’s perceptions of their ERS practices. Research examining ethnic-racial socialization (ERS) rarely employs dyadic designs that capture both parent and youth perspectives, and even fewer studies consider both ERS beliefs and enacted behaviors. Distinguishing between beliefs and behaviors is essential, as caregivers’ intentions may not always translate into observable practices, and youth may interpret these messages in ways that differ from what was intended. Our findings suggest there is specificity in the relationship between parental ERS beliefs and youth’s perceptions of their practices. Our finding that preparation for bias and promotion of mistrust beliefs informed these specific behaviors is consistent with extant literature (Witherspoon et al., 2021), as well as notions posited by the integrative model (García Coll et al., 1996) that minoritized families may develop an adaptive culture, which then informs their subsequent behaviors with their children. However, we found that greater cultural-egalitarianism beliefs were associated with less perceived cultural-egalitarianism practices from youth. This finding is contrary to previous research that has demonstrated a significant association between parents’ cultural socialization attitudes and their enacted behaviors with both adolescents and young children (Derlan et al., 2016; D. Hughes et al., 2008). One reason could be that we are measuring both cultural socialization and egalitarianism together. Another reason could be that youth are active agents in the ERS process, so while parents may strongly endorse cultural-egalitarianism beliefs, they may not explicitly communicate or model those beliefs in ways that adolescents recognize as cultural-egalitarianism practices (D. Hughes et al., 2006; Smith-Bynum, 2023). Thus, youth may not perceive the intended messages, or they may interpret them differently than parents expect.

Additionally, in Latino families, preparation for bias beliefs was associated with more perceived cultural-egalitarianism practices, and promotion of mistrust beliefs was associated with more perceived preparation for bias practices. As described above, in a new destination context, Latino parents who endorse preparation for bias beliefs may engage in ERS practices that both promote equality and respect for all groups while also affirming the child’s own ethnic identity (Smith-Bynum, 2023). While this pattern has been primarily documented in Black families, the current findings suggest that it is an important ERS processes among Latino families—particularly in contexts where cultural integration and the threat of discrimination coexist. In the same vein, belief in the importance of promotion of mistrust beliefs may engender both promotion of mistrust and preparation for bias as protective strategies to cope with racial discrimination and possibly negative intergroup contact. Including how specific ERS beliefs relate to specific practices is important because it highlights the specificity of these relationships, revealing how distinct parental ERS beliefs (e.g., preparation for bias, egalitarianism, promotion of mistrust) guide targeted forms of socialization. This approach moves beyond global measures of ERS to uncover nuanced pathways through which beliefs translate into concrete practices, offering clearer insight into how and why certain messages are delivered to youth.

We also found that ERS beliefs were directly related to youth’s ERI, and that ethnic-racial group moderated these associations. As expected, preparation for bias beliefs was associated with less public regard. Preparation for bias beliefs may be associated with lower public regard in youth because these messages often communicate the expectation that others will view them negatively or treat them unfairly based on their race or ethnicity, which may lead youth to internalize the idea that society holds negative views of their ethnic-racial group, thereby reducing their perception that others view their group positively (D. Hughes et al., 2006; Neblett et al., 2009).

Parents’ promotion of mistrust beliefs was associated with less centrality and private regard in Latino families and more centrality in Black families. This discrepancy in findings could be due to the new destination context, where Latino youth frequently interact with Black neighbors and peers in school and other social environments. Due to their frequent interactions, they may perceive parental caution regarding interracial interactions as unjust or racially biased (Daga & Raval, 2018; Woo et al., 2020), which can inadvertently prompt them to distance themselves from their ethnic-racial group, contrary to parents’ intentions. Additionally, Latino youth who receive messages that they need to be wary of other ethnic-racial groups may form “colorblind” relationships or friendships that deemphasize race in order to decrease the possibility of experiencing discrimination or negative interactions. However, such relationships may limit adolescents’ ability to internalize their ethnic-racial identity as a meaningful and central part of who they are. In addition, Latino parents’ promotion of mistrust beliefs may result from their youth’s ethnic-racial discrimination experiences, and discrimination is linked to lower positive ethnic-racial affect in Latino youth (Stein et al., 2023; Wang & Yip, 2020). Meanwhile, for Black youth, who are the numerical majority in this context, promotion of mistrust may contribute to heightened intergroup hostility and anxiety (Daga & Raval, 2018) and may lead them to socially isolate from their non-Black peers (Atkin et al., 2018).

These differences can also be understood through the lens of racial coping strategies. Hamm and Coleman (2001) identified three interpersonal coping strategies that adolescents use to navigate diverse school environments: multicultural, separation, and assimilation/acculturation. For Latino youth, living in predominantly Black neighborhoods may encourage more assimilation/acculturation coping, which implies that they should sacrifice their ethnic cultural values to avoid rejection from the dominant group (Neblett et al., 2009; Rivas-Drake et al., 2009). Meanwhile, Black youth in a Black-majority environment may be more likely to adopt strategies of separation or multicultural coping, making their ERI a central aspect of their self-concept. The importance of neighborhood context could explain the discrepant findings in the literature where the promotion of mistrust has been link to both negative (Dunbar et al., 2015) and positive (Nelson et al., 2018) youth adjustment.

### 4.2. Neighborhood Factors, ERS, and Youth’s Self-System

Neighborhoods are both inhibiting and promoting environments for families. As hypothesized, for these Black and Latino families, greater neighborhood disadvantage was associated with less belief in the importance of preparation for bias and promotion of mistrust. For parents in disadvantaged neighborhoods, in which neighborhood resources are limited, preparing their children for discrimination may be less important than meeting their needs. Furthermore, due to the increased levels of environmental and interpersonal stressors associated with living in disadvantaged neighborhoods (G. W. Evans, 2004; Leventhal & Brooks-Gunn, 2000), parents might feel that discrimination and bias are inevitable, which can result in a lower sense of efficacy in their ability to prepare their children to cope with discrimination and thus less belief that it is an important parenting practice (Anderson & Stevenson, 2019). According to Racial Encounter Coping Appraisal and Socialization Theory (RECAST), this diminished efficacy may reflect feelings of helplessness in the face of racism and a lack of resources to effectively engage in preparation for bias socialization (Anderson & Stevenson, 2019). Neighborhood disadvantage is well-established as relating to less parental self-efficacy (Jones & Prinz, 2005), and this study suggests that it extends to self-efficacy beliefs about culturally specific parenting practices.

There were ethnic-racial differences in the relation of neighborhood disadvantage to youth’s self-esteem and ERI. Consistent with our predictions and prior research (Bámaca et al., 2005; Behnke et al., 2011; White et al., 2018), Latino youth in disadvantaged neighborhoods reported lower self-esteem and all aspects of ERI. However, for Black youth, while neighborhood disadvantage was associated with lower self-esteem and public regard, as expected, it was also associated with higher centrality. This differs from past research, which has found no relationship, or a negative relationship (Chavous et al., 2003; Rivas-Drake & Witherspoon, 2013), as seen in Latino youth. For Black families, increased neighborhood disadvantage was correlated with higher neighborhood diversity, meaning a greater concentration of Latino neighbors. In disadvantaged neighborhoods with increased diversity, particularly with more Latino families, Black youth may become more aware of their racial identity in relation to other ethnic-racial groups, making race a more salient social category. Another explanation is that in disadvantaged neighborhoods, the increased presence of another ethnic-racial group can lead to a perception of competition for limited resources, as different groups vie for access to economic, social, and educational opportunities (Jencks & Mayer, 1990). This perceived competition can make racial identity more central, as youth may turn to their ERI for solidarity and strength in navigating the challenges of resource scarcity (Phinney, 1990; Sellers et al., 2006). In line with this finding, we found that neighborhood diversity was associated with greater centrality for Black youth.

Neighborhood diversity was also associated with parental belief in the importance of promotion of mistrust for all families and greater self-esteem and centrality for Latino youth. Our finding with neighborhood diversity and greater belief in the promotion of mistrust differs from prior work with Black families (Caughy et al., 2006), but this discrepancy might reflect the historically tense relationships between Black and Latino adults in new destination contexts (e.g., Brown & Brooks, 2006). Black and Latino parents may be more likely believe it is important to emphasize caution and mistrust in their ERS practices, aiming to prepare their children for possible discrimination or negative interactions with members of other ethnic-racial groups. Neighborhoods with greater diversity can also expose minoritized groups to more frequent experiences of discrimination, even from other ethnic-racially minoritized groups (English et al., 2014). These experiences may increase perceptions that those outside of their ethnic-racial group are not to be trusted or may harbor prejudices, which could heighten beliefs that it is important for their children to approach others with caution.

In Latino families, neighborhood diversity was associated with greater self-esteem and centrality. For this study, greater diversity was synonymous to greater concentration of Latino neighbors. This is in line with research that shows that higher concentrations of neighbors with similar ERIs is beneficial for Latino youth’s development of self (Pasco et al., 2021b). Neighborhood diversity predicted ethnic-racial centrality, suggesting that students living with more co-ethnics view being Latino as more central to their sense of self. Living in neighborhoods with greater concentrations of Latinos may encourage the development and articulation of ERI-centered attitudes. These environments often provide supportive conditions for the expression of Latino culture (Witherspoon et al., 2023), which is frequently subject to scrutiny, marginalization, or commodification within mainstream societal contexts.

Neighborhood cohesion was the only neighborhood-level factor associated with belief in the importance of cultural-egalitarianism and preparation for bias. This is in line with previous work (Pasco et al., 2021a; Saleem et al., 2016). As stated above, previous research has shown that parents who perceive strong social support and reside in neighborhoods characterized by positive social dynamics are more likely to report a greater sense of parental efficacy (Izzo et al., 2000), which may influence parents’ ERS efforts (Caughy et al., 2006). Parents have greater self-efficacy in their ability to foster pride and knowledge of their ethnic-racial background and prepare their children to cope with discrimination and thus in their belief that these are important parenting practices.

### 4.3. Parental Cultural Factors, ERS, and Youth’s Self-System

One goal of this paper was to explore whether parental discrimination and ERI were related to ERS beliefs and youth self-system in the face of neighborhood-level factors. Contrary to our hypotheses, parental discrimination and ERI were unrelated to parental ERS beliefs, except for parental discrimination being marginally related to greater belief in the importance of promotion of mistrust. Scholars have theorized that parents’ own racial discrimination experiences and ERI influence their culture-related parenting practices (Umaña-Taylor & Hill, 2020) due to the fact parenting does not occur in a vacuum but is shaped by parents’ lived experiences and self-perceptions. However, empirical research in this area is mixed. For instance, earlier research found that parents who experienced unfair treatment were more likely to engage in preparation for bias socialization (D. Hughes et al., 2006). Yet, studies with Latino families have not consistently found this association. Empirical research has found no significant associations between Latino parents’ discrimination experiences and preparation for bias messages, despite being theorized (Espinoza et al., 2016; Kulish et al., 2019). More recent studies that incorporate neighborhood characteristics further complicate the picture. For example, Witherspoon et al. (2022), with a sample of Black and Latino families, found no significant relationships between parental discrimination, their ERI, and their ERS when neighborhood context was considered (Witherspoon et al., 2022). Therefore, although we expected parents’ cultural factors to influence their parenting beliefs and practices, our findings, in line with current research, suggest that individual-level cultural factors may not directly influence Black and Latino parents’ ERS beliefs when accounting for neighborhood context. Alternatively, the relationship between parents’ own cultural assets (e.g., ERI) or stressors (e.g., discrimination) and their attitudes about ERS may be more complex and nuanced, with neighborhood characteristics moderating these associations. For example, Saleem et al. (2016) found that the relationship between parents’ experiences of racial discrimination and their ERS messages varied depending on the level of neighborhood cohesion. Similarly, Witherspoon et al. (2022) found that parents’ ethnic-racial affirmation was more strongly linked to ERS messages in lower socioeconomic (SES) neighborhoods compared to higher SES ones.

As predicted, parental discrimination was associated with greater centrality for all youth and less public regard for Black youth. In contrast, parental ERI was associated with greater public regard. Current theories of ERI development suggest that centrality and public regard are largely informed by contextual factors and identity-relevant experiences in adolescence (Sellers et al., 1998; C. D. Williams et al., 2020). Thus, vicariously experiencing racial discrimination through parental discrimination experiences may carry influential messages about how others disvalue one’s group and increase awareness of societal stereotypes (Rivas-Drake et al., 2009). This would make one’s ethnic-racial group more central to their sense of self and decrease their belief that others perceive their racial-ethnic group positively. In the same vein, when parents have a strong positive ERI and actively promote pride in their heritage, it can lead to higher public regard among their children (Priest et al., 2014).

### 4.4. Mediation

The mediation of neighborhood and parental cultural factors through ERS beliefs, and sequentially through ERS beliefs and practices, suggests interesting propositions when we investigate the factors at play. We focus on total indirect effects and specific indirect effects differing across ethnic-racial groups. Neighborhood disadvantage lessened parents’ belief in the importance of preparation for bias and promotion of mistrust. As expected, these lessened ERS beliefs led to neighborhood disadvantage being indirectly related to lower ERI for youth in Black families. For Black families, preparation for bias and promotion of mistrust are key protective strategies that help Black youth navigate a racially hostile society. As such, less belief in their importance may signal a lack of racial preparation, potentially leaving youth feeling unarmed or disconnected from their racial identity, thus contributing to weaker ERI development. However, in Latino families, there was no total indirect effect because neighborhood disadvantage was associated with less promotion of mistrust beliefs, and that lower promotion of mistrust predicted stronger ERI.

In new destinations, when neighborhood disadvantage lessens parents’ mistrust beliefs, youth may feel more autonomy to engage in cultural exploration, seek out community connections, and define their ethnic identity in empowering ways. This aligns with research indicating that promotion of mistrust may, in some contexts, limit ERI exploration and peer interactions (Derlan et al., 2016), while openness supports positive ERI development. Neighborhood diversity increased parents’ belief in the importance of promotion of mistrust. Unlike disadvantage, this greater ERS belief led to neighborhood diversity being indirectly related to lower self-esteem, centrality, and private regard in Latino youth, while in Black youth, it was indirectly associated with greater private regard. The discrepant impact of ERS beliefs between Black and Latino families, and difference from neighborhood disadvantage and diversity, underscore the importance of contextualizing ERS not only by neighborhood characteristics but also by group-specific histories and meanings attributed to racial messages.

Neighborhood cohesion increased parents’ belief in the importance of cultural-egalitarianism and preparation for bias; however, the indirect effects through these beliefs had distinct and, at times, divergent implications for Black and Latino youth. Unexpectedly, this greater belief in cultural-egalitarianism lead to neighborhood cohesion being indirectly related to lower self-esteem among all youth and lower centrality and private regard in Latino youth via reduced perceptions of cultural-egalitarianism practices. In contrast, increased parental belief in the importance of preparation for bias in cohesive neighborhoods had more uniformly positive outcomes. Neighborhood cohesion through this greater belief was associated with higher public regard across all youth and increased centrality among Black youth, and through increased beliefs, which in turn increased cultural-egalitarianism practices with greater self-esteem, private regard, and centrality among Latino youth. Overall, neighborhood cohesion indirectly contributed to enhanced self-esteem among Latino youth and heightened centrality among Black youth, as expected.

The discrepant findings regarding the indirect effects of neighborhood cohesion on youth’s self-system, mediated through increased cultural-egalitarianism and preparation for bias beliefs, highlights the complex interplay between ERS beliefs and practices. Specifically, these results were driven by the fact that cultural-egalitarianism beliefs paradoxically diminished youth’s perception of the frequency of these practices, while preparation for bias beliefs increased these practices. Furthermore, these dynamics are particularly salient among Latino youth, where cultural-egalitarianism practices are associated with higher centrality and private regard, underscoring the nuanced role of ERS practices in identity development. This underscores the necessity of examining both ERS beliefs and practices as distinct yet interconnected mechanisms through which neighborhood characteristics influence Black and Latino youth’s self-systems. Further research is imperative to elucidate how cultural socialization and egalitarianism beliefs and practices interact and whether this idea of cultural-egalitarianism is a particular ERS practice that is distinct from these types of beliefs, particularly in diverse ecological contexts.

Lastly, parental discrimination indirectly impacted Black and Latino youth’s private regard through promotion of mistrust beliefs in opposite ways. Parental experiences of discrimination may indirectly shape Black and Latino youth’s private regard by promoting mistrust beliefs, though this influence operates in distinct ways across these groups, particularly in new destination contexts. For Black youth, increased ERS beliefs around mistrust can reinforce group solidarity, as cautionary messages about the challenges of racism help to foster a strong sense of pride and collective identity. In this context, mistrust toward outsiders may serve as a protective mechanism, enhancing private regard and ingroup identification. In contrast, for Latino youth, the promotion of mistrust can have more negative effects. Given the ambiguity surrounding their racial or ethnic classification in new destinations—coupled with the additional layers of stigma related to immigrant status or language barriers—mistrust messages may foster alienation rather than solidarity. This can lead to a weakened sense of connection to both their own ethnic group and the broader society, ultimately lowering private regard. These findings underscore the importance of considering the ways in which parental discrimination shapes youth ERI differently for Black and Latino adolescents, particularly in environments where ethnic communities are newly established and face unique challenges.

### 4.5. Limitations and Future Directions

This study broadens current understandings of ERS by examining how parental cultural beliefs and neighborhood conditions work in tandem to shape Black and Latino adolescents’ self-systems (self-esteem and ERI). By situating ERS within both family and neighborhood contexts, our findings highlight the layered ways in which environment informs parenting and developmental processes. At the same time, several limitations—namely the scope of neighborhood factors, role of gender, and other parenting dimensions—temper the conclusions that can be drawn. These limitations, in addition to methodological considerations related to measurement reliability, sample size, and study design, point to directions for future research on how neighborhood, family, and cultural contexts jointly shape adolescents’ self-systems.

First, this study was restricted in the scope of neighborhood factors it could assess. Beyond ethnic-racial diversity, future work should account for concentration effects—whether a co-ethnic group or majority group holds numerical dominance in a neighborhood. Ethnic-racial concentration may carry distinct implications for how parents socialize their youth around ethnicity and race (White et al., 2018). This may be especially relevant in new destination contexts, where families navigate unique demographic shifts and intergroup dynamics (Massey, 2008). A fuller picture of neighborhood context would provide a more nuanced understanding of how diversity and concentration jointly shape parenting beliefs and strategies related to youth’s self-system.

Second, future research should examine how gender alongside race shapes ERS processes and adolescent development. Prior studies underscore the need for a gender-sensitive lens by documenting gendered differences in these associations among Black and Latino families (Dunbar et al., 2022; Kulish et al., 2019). Gendered parenting beliefs may be particularly important for understanding how parents deliver ERS messages across neighborhood contexts. Preliminary evidence suggests that gender role socialization can moderate the association between neighborhood context and adolescents’ ERI. For example, among Latino parent–adolescent dyads from this sample, adolescents whose parents endorsed more traditional gender role beliefs reported lower ERI when neighborhood problems were higher (Goldstein et al., 2025). These associations did not emerge for neighborhood connectedness and, importantly, were consistent across Latino youth. Thus, moving forward, it will be important for research to conceptualize gender not only as an identity category (e.g., being a boy vs. being a girl) but also as a cultural context that structures parenting beliefs, socialization practices, and youth self-system development.

Third, ERS was examined in relative isolation from the broader parent–adolescent relationship. At the same time, the current study’s inclusion of both adolescent and parent perspectives is a notable strength because it provides a richer understanding of ERS processes. Future work could extend this approach by incorporating other socializing figures—such as extended family members, teachers, schools, and peers—who play critical roles in reinforcing or challenging parental ERS messages across contexts (Kulish et al., 2019; Umaña-Taylor & Hill, 2020). Moreover, ERS is only one aspect of parenting and is deeply interconnected with other practices such as monitoring, control, and relationship quality (e.g., warmth; N. A. Smith et al., 2022). Employing person-centered approaches, such as profile analyses, could help to identify patterns of parenting practices and clarify how these jointly shape Black and Latino youths’ self-systems (Kim et al., 2019; Witherspoon et al., 2019).

Finally, several methodological considerations limit the conclusions that can be drawn from this study. A primary limitation of this study is the relatively small sample size, which constrained statistical power to detect small to moderate effects. Power analysis with Monte Carlo simulations indicated that a substantially larger sample size (N = 736) would be necessary to achieve adequate power (≥0.80) for significant model parameters. Additionally, these simulations also suggested that a few non-significant findings may reflect Type II errors rather than true null effects. As such, the lack of association between ERS beliefs and practices as well as cultural-egalitarianism and centrality and private regard in Black youth should be interpreted with caution. However, achieving such a large sample was not feasible given the mixed-methods design, which included focus group participation and required substantial time and engagement from participants. Taken together, these factors necessitate caution in interpreting null results and underscore the importance of replicating these findings in larger, possibly multi-site or collaborative studies to increase statistical power and generalizability. Second, the ERI measures demonstrated relatively low reliability, which—although comparable to other studies with Black (Byrd & Chavous, 2011) and Latino (Kulish et al., 2019) youth—may have impacted the stability of the findings. Third, the cross-sectional design precludes inferences about directionality. Future studies with larger, longitudinal samples are needed to track how parenting beliefs, youth perceptions, and neighborhood context change over time. Such work would clarify whether these associations are unidirectional or bidirectional (Contreras et al., 2021) and help to identify critical developmental periods in which culturally informed parenting practices are most effective in supporting Black and Latino adolescents’ self-systems.

### 4.6. Policy and Practical Implications

Self-esteem and ethnic-racial identity (ERI) play a critical role in supporting positive development for racially and ethnically minoritized youth. Our findings reaffirm that parents’ ethnic-racial socialization (ERS) beliefs and practices shape youth’s identity development and self-concept, aligning with prior work among Black and Latino families (D. Hughes et al., 2008). Importantly, this research highlights the need to position parents as key agents in youth identity formation, underscoring the importance of engaging caregivers in interventions that foster healthy ERI and self-esteem.

From a practice standpoint, these findings suggest that interventions aimed at enhancing adolescent self-esteem and ERI would benefit from directly incorporating components that target parental ERS. Programs should provide structured guidance to help parents initiate and sustain developmentally appropriate conversations about race, ethnicity, and discrimination. ERS interventions such as those proposed by Anderson et al. (2018) and racial socialization programs for Black families and emerging adaptations for Latino populations (Stein et al., 2021; M. Evans & McDonald, 2023) demonstrate promising directions. Scaling such efforts across racially diverse communities could meaningfully support youth development.

Beyond the family context, this study underscores the significance of neighborhood environments in shaping both parental beliefs and youth outcomes. Structural and social neighborhood characteristics influenced parents’ valuation of ERS, which in turn impacted youth’s perceptions and their internal self-system. These findings reinforce ecological frameworks of development and point to the need for a dual focus on both micro-level (e.g., family) and macro-level (e.g., community) influences. Given the persistent residential segregation experienced by many Black and Latino families, place-based disparities must be recognized not only as social injustices but also as developmental risk factors that constrain parenting practices and identity development.

Thus, there is a clear policy imperative to support neighborhood conditions that affirm cultural identity, promote social cohesion, and reduce exposure to racial discrimination. Community-based programs that facilitate intergroup engagement, celebrate cultural heritage, and cultivate inclusive neighborhood norms can serve as protective mechanisms for youth. Initiatives such as Your Family, Your Neighborhood (Lechuga-Peña & Brisson, 2018) represent scalable models that embed family support within community development efforts. Policymakers should consider investments in these integrative, neighborhood-centered programs as part of broader strategies to address racial inequities in health, education, and youth development.

Finally, our findings reveal the need for greater attention to how context—especially structural inequalities tied to race and place—shapes parenting and adolescent development. Future research and policy should move beyond individual-level interventions and toward multilevel approaches that simultaneously address family dynamics and systemic factors. Equitable urban planning, housing access policies, and community infrastructure investments must be viewed as critical components of developmental support systems for minoritized youth. Interventions focused on cultural identity, acknowledging contextual constraints, and empowering both parents and youth hold the potential to foster more just and affirming environments in which all adolescents can thrive.

## 5. Conclusions

This study found that parents’ ethnic-racial socialization (ERS) beliefs and practices, shaped by both neighborhood and parental cultural assets and stressors, play a meaningful role in shaping adolescents’ self-esteem and ethnic-racial identity (ERI). These findings emphasize the importance of considering the broader ecological context in which ERS occurs, particularly for Black and Latino families living in new destination communities. Supporting youth self-esteem and ERI requires not only culturally responsive parenting interventions but also policies and programs that address neighborhood-level factors, such as social cohesion and structural inequality. A contextualized approach to identity development is essential for promoting positive outcomes among racially and ethnically minoritized youth.

## Figures and Tables

**Table 1:** Ethnic-racial group differences in demographic characteristics and study variables.

	Latino (N = 95)	Black (N = 89)	t or χ^2^ (df)
Variable	Frequency (Valid %) or Mean (SD)		
Demographics			
Parent			
Age	39.81 (8.88)	41.52 (9.44)	−1.03 (120)
Marital status			15.1 *** (2)
Not married or cohabiting	27.3% ^a^	59.4% ^b^	
Married or cohabitating	39.0%	25.0%	
Separated/divorced/widowed	33.8% ^a^	15.6% ^b^	
Educational level			15.48 *** (3)
Less than high school	27.4% ^a^	3.2% ^b^	
High school degree	38.4%	43.5%	
Some postsecondary education	23.3% ^a^	40.3% ^b^	
Bachelor’s degree or higher	11.0%	12.9%	
Family income			
Less than USD 10,000	44.6%	30.4%	15.57 ** (4)
USD 10,001–20,000	33.9% ^a^	14.3% ^b^	
USD 20,001–30,000	8.9%	17.9%	
USD 30,001–40,000	3.6% ^a^	19.6% ^b^	
More than USD 41,000	8.9%	17.9%	
Family income-to-needs ratio	0.74 (0.91)	1.20 (1.12)	−2.32 * (120)
Years living in current neighborhood	7.21 (6.77)	10.5 (10.23)	−2.14 * (120)
Adolescent			
Age	13.33 (1.95)	13.44 (1.87)	−0.42 (182)
Gender (% female)	55.4%	59.8%	−0.58 (177)
Study Variables			
Neighborhood disadvantage	−0.11 (0.85)	0.12 (0.74)	−1.95 ^+^ (182)
Neighborhood diversity	0.57 (0.11)	0.57 (0.12)	−0.35 (182)
Neighborhood cohesion	2.35 (0.93)	2.69 (0.66)	−2.88 ** (182)
Parental discrimination	2.03 (1.04)	1.95 (0.76)	0.65 (178)
Parental ethnic-racial identity	3.60 (0.99)	3.70 (0.86)	−0.62 (135)
Cultural-egalitarianism beliefs (PR)	3.59 (0.51)	3.57 (0.60)	−0.28 (178)
Preparation for bias beliefs (PR)	3.47 (0.66)	3.62 (0.53)	−1.53 (140)
Promotion of mistrust beliefs (PR)	3.02 (0.96)	2.14 (0.91)	6.33 *** (178)
Cultural-egalitarianism practices (YR)	2.28 (0.49)	2.43 (0.43)	−2.08 * (145)
Preparation for bias practices (YR)	1.66 (0.48)	1.66 (0.42)	0.11 (145)
Promotion of mistrust practices (YR)	1.13 (0.33)	1.20 (0.51)	−0.90 (145)
Youth self-esteem	1.95 (0.53)	2.23 (0.57)	−3.24 *** (165)
Youth centrality	3.89 (0.88)	4.02 (0.75)	−1.07 (178)
Youth private regard	4.22 (0.69)	4.35 (0.61)	−1.28 (178)
Youth public regard	3.42 (0.73)	3.19 (0.92)	1.79 ^+^ (178)

Note: Mean difference tests reported with *t*-test and chi-square statistics. For Chi-Square tests, means that do not share superscripts differ *p* < 0.05; post hoc test is Bonferroni. PR = Parent report, YR = Youth report. ^+^ *p* < 0.10; * *p* < 0.05; ** *p* < 0.01; *** *p* < 0.001.

**Table 2:** Bivariate correlations of study variables.

Variable	1	2	3	4	5	6	7	8	9	10	11	12	13	14	15	16	17
1. Adolescent Age	--	−0.03	−0.15	0.07	0.07	−0.12	−0.01	−0.07	0.04	−0.03	0.17	0.04	−0.12	−0.09	0.14	0.17	−0.10
2. Family Income	0.25 *	--	−0.29 *	−0.06	0.19	0.28 *	0.15	−0.04	0.10	−0.29 *	0.22	0.07	−0.02	0.21	0.02	0.13	0.09
3. Neighborhood Disadvantage	0.18	−0.18	--	0.32 **	−0.06	0.16	−0.05	−0.03	−0.19	−0.09	−0.16	0.09	0.10	−0.26 *	−0.22 *	−0.27 **	−0.28 **
4. Neighborhood Diversity	0.02	−0.07	0.22 *	--	0.06	−0.19	−0.24 *	0.12	−0.01	0.12	0.03	0.04	−0.03	0.22 *	−0.01	−0.06	−0.12
5. Neighborhood Cohesion	−0.08	0.05	−0.03	0.23 *	--	−0.05	0.00	0.29 **	0.27 **	0.07	0.23 *	0.24 *	0.14	−0.12	0.01	0.17	−0.03
6. Parental Discrimination	−0.09	−0.16	0.01	−0.13	0.01	--	−0.25 *	−0.27 **	−0.16	0.09	−0.18	0.11	0.06	−0.23 *	0.04	−0.02	−0.14
7. Parental Ethnic-Racial Identity	−0.13	0.07	0.01	0.18	0.12	−0.06	--	0.14	0.12	−0.09	0.15	−0.01	−0.07	−0.03	0.01	0.04	0.21 *
8. Cultural-Egalitarianism Beliefs (PR)	−0.37 **	0.10	−0.16	0.06	0.22 *	0.11	0.17	--	0.72 **	0.39 **	0.20	0.10	−0.04	0.14	−0.13	−0.01	0.11
9. Preparation for Bias Beliefs (PR)	−0.12	0.18	−0.11	0.02	0.27 *	0.13	0.03	0.68 **	--	0.31 **	0.42 **	0.27 *	−0.06	0.16	0.00	0.14	0.15
10. Promotion of Mistrust Beliefs (PR)	−0.17	0.00	−0.16	0.21	0.14	0.18	−0.07	0.32 **	0.32 **	--	−0.03	0.20	0.16	−0.10	−0.22 *	−0.19	0.06
11. Cultural-Egalitarianism Practices (YR)	0.07	−0.02	0.19	0.06	−0.12	0.08	0.07	−0.25	−0.16	0.04	--	0.29 **	−0.14	0.26 *	0.42 **	0.47 **	0.08
12. Preparation for Bias Practices (YR)	0.07	0.20	0.13	0.04	0.01	0.09	−0.19	−0.18	−0.11	−0.22	−0.01	--	0.20	−0.16	0.04	0.02	−0.27 *
13. Promotion of Mistrust Practices (YR)	0.04	−0.27	0.17	0.11	−0.12	0.10	−0.10	−0.29 *	−0.32 *	−0.12	−0.27 *	0.21	--	−0.30 **	−0.13	−0.15	−0.08
14. Youth Self-Esteem	0.09	0.21	−0.14	−0.06	0.06	0.14	0.05	0.12	0.21	0.13	0.14	−0.08	−0.31 *	--	0.26 *	0.32 **	0.20
15. Youth Centrality	0.09	0.01	0.17	0.25 *	0.20	0.09	0.05	0.12	0.24 *	0.18	−0.09	0.08	−0.06	0.15	--	0.71 **	0.03
16. Youth Private Regard	0.07	0.01	0.09	0.05	0.11	0.11	−0.08	0.13	0.23 *	0.27 *	−0.13	0.09	−0.07	0.22	0.63 **	--	0.17
17. Youth Public Regard	−0.06	0.11	−0.20	0.02	−0.11	−0.40 **	0.21	−0.02	0.11	0.08	0.20	−0.27 *	−0.24	0.29 *	−0.03	−0.08	--

Note: Correlations above the diagonal are for Latino families, below the diagonal are for Black families. PR = Parent report, YR = Youth report, * *p* < 0.05.; ** *p* < 0.01.

**Table 3:** Model fit indices and chi-square differences for multigroup models by outcome.

Model	Pathway Freed	χ^2^	*df*	CFI	TLI	SRMR	RMSEA (90% CI)	∆χ^2^ from Previous Models	∆*df*
Self-Esteem									
1. Unconstrained		80.29	70	0.96	0.93	0.07	0.02 (0.00, 0.08)		
2. Constrained		135.17	105	0.89	0.86	0.10	0.06 (0.02, 0.08)	54.87 ***	35
3. Model 1	PFB Beliefs (PR) → CSEgal Practices (YR)	118.48	104	0.95	0.93	0.09	0.04 (0.00, 0.07)	16.69 ***	1
4. Model 2	PMT Beliefs (PR) → PFB Practices (YR)	112.13	103	0.97	0.96	0.09	0.03 (0.00, 0.06)	6.35 *	1
5. Model 3	N Disadvantage → Self-Esteem	108.14	102	0.98	0.97	0.09	0.03 (0.00, 0.61)	3.99 *	1
Centrality									
1. Unconstrained		73.00	70	0.99	0.98	0.07	0.02 (0.00, 0.07)		
2. Constrained		148.74	105	0.84	0.79	0.10	0.07 (0.04, 0.09)	75.74 ***	35
3. Model 1	PFB Beliefs (PR) → CSEgal Practices (YR)	132.43	104	0.90	0.86	0.10	0.06 (0.02, 0.08)	16.31 ***	1
4. Model 2	CSEgal Practices (YR) → Centrality	120.43	103	0.94	0.92	0.09	0.04 (0.00, 0.07)	12.00 **	1
5. Model 3	N Disadvantage → Centrality	113.90	102	0.96	0.94	0.09	0.04 (0.00, 0.07)	6.54 *	1
6. Model 4	PMT Beliefs (PR) → Centrality	106.44	101	0.98	0.97	0.08	0.02 (0.00, 0.06)	7.46 **	1
7. Model 5	PMT Beliefs (PR) → PFB Practices (YR)	99.98	100	1.00	1.00	0.08	0.00 (0.00, 0.06)	6.46 *	1
8. Model 6	PFB Beliefs (PR) → Centrality	95.17	99	1.00	1.00	0.08	0.00 (0.00, 0.05)	4.81 *	1
Private Regard									
1. Unconstrained		72.31	70	0.99	0.98	0.07	0.02 (0.00, 0.06)		
2. Constrained		146.50	105	0.85	0.80	0.11	0.07 (0.04, 0.09)	74.19 ***	35
3. Model 1	PFB Beliefs (PR) → CSEgal Practices (YR)	130.18	104	0.91	0.87	0.10	0.05 (0.01, 0.08)	16.32 ***	1
4. Model 2	CSEgal Practices (YR) → Private Regard	118.27	103	0.95	0.93	0.09	0.04 (0.00, 0.07)	11.91 **	1
5. Model 3	PMT Beliefs (PR) → Private Regard	110.20	102	0.97	0.96	0.09	0.03 (0.00, 0.06)	8.07 **	1
6. Model 4	N Disadvantage → Private Regard	102.06	101	1.00	1.00	0.08	0.01 (0.00, 0.06)	8.14 **	1
7. Model 5	PMT Beliefs (PR) → PFB Practices (YR)	95.82	100	1.00	1.00	0.08	0.00 (0.00, 0.05)	6.24 *	1
Public Regard									
1. Unconstrained		73.93	70	0.99	0.97	0.07	0.03 (0.00, 0.07)		
2. Constrained		136.08	105	0.89	0.86	0.10	0.06 (0.02, 0.08)	62.14 **	35
3. Model 1	PFB Beliefs (PR) → CSEgal Practices (YR)	119.85	104	0.95	0.93	0.09	0.04 (0.00, 0.07)	16.23 **	1
4. Model 2	P Discrimination → Public Regard	107.19	103	0.99	0.98	0.08	0.02 (0.00, 0.06)	12.66 **	1
5. Model 3	PMT Beliefs (PR) → PFB Practices (YR)	101.16	102	1.00	1.00	0.08	0.00 (0.00, 0.05)	6.02 *	1

Note: Fit indices include Comparative Fit Index (CFI), Tucker–Lewis Index (TLI), Standardized Root Mean Square Residual (SRMR), and Root Mean Square Error of Approximation (RMSEA). Chi-square difference tests (∆χ^2^) compare each model to the previous one in the sequence. * *p* < 0.05, ** *p* < 0.01, *** *p* < 0.001. CSEgal = Cultural-Egalitarianism, PFB = Preparation for Bias, PMT = Promotion of Mistrust; N Disadvantage = Neighborhood Disadvantage, P Discrimination = Parental Discrimination; PR = Parent-Report, YR = Youth Report.

**Table 4:** Standardized indirect and total indirect effects of neighborhood and parental factors on youth outcomes, with 95% confidence intervals (significant pathways only).

Variables	Model 1: Self-Esteem		Model 2: Centrality		Model 3: Private Regard		Model 4: Public Regard	
	Latino	Black	Latino	Black	Latino	Black	Latino	Black
	B [95% CI]		B [95% CI]		B [95% CI]		B [95% CI]	
**N Disadvantage →**								
PFB Beliefs (PR)	−0.01 [−0.06, 0.004]		0.02 [−0.01, 0.08]	**−0.03 [−0.11, −0.002] _a_**	−0.01 [−0.06, 0.01]		**−0.03 [−0.10, −0.004] _a_**	
PMT Beliefs (PR)	0.02 [−0.003, 0.06]		**0.05 [0.01, 0.13] _a_**	−0.02 [−0.09, 0.02]	**0.05 [0.01, 0.12] _a_**	**−0.05 [−0.12, −0.01] _a_**	**−0.03 [−0.10, 0.003] _b_**	
PFB (PR) → CSEgal (YR)	**−0.01 [−0.04, −0.001] _a_**	0.001 [−0.002, 0.10]	**−0.04 [−0.12, −0.01] _a_**	−0.001 [−0.02, 0.002]	**−0.03 [−0.08, −0.01] _a_**	−0.001 [−0.02, 0.002]	**−0.01[−0.04, 0.00] _b_**	0.001 [−0.002, 0.01]
PFB (PR) → PFB (YR)	0.003 [0.00, 0.02]		−0.002 [−0.02, 0.002]		0.00 [−0.01, 0.004]		**0.01 [0.001, 0.04] _a_**	
PMT (PR) → PFB (YR)	0.004 [0.00, 0.02]	−0.003 [−0.02, 0.001]	−0.002 [−0.02, 0.01]	0.002 [−0.004, 0.02]	0.00 [−0.01, 0.01]	0.00 [−0.01, 0.01]	**0.01 [0.002, 0.04] _a_**	−0.01 [−0.04, 0.002]
PMT (PR) → PMT (YR)	**0.004 [0.001, 0.02]_a_**		0.003 [−0.001, 0.02]		0.002 [0.00, 0.01]		0.001 [−0.01, 0.01]	
Total Indirect	0.004 [−0.03, 0.05]	0.01 [−0.03, 0.05]	0.05 [−0.01, 0.13]	**−0.05 [−0.13, −0.002] _a_**	0.02 [−0.03, 0.09]	**−0.06 [−0.13, −0.02] _a_**	−0.04 [−0.10, 0.01]	**−0.05 [−0.12, −0.01] _a_**
**N Diversity →**								
PMT Beliefs (PR)	−0.12 [−0.36, 0.03]		**−0.33 [−0.80, −0.04] _a_**	0.14 [−0.13, 0.55]	**−0.30 [−0.69, −0.07] _a_**	**0.31 [0.07, 0.72] _a_**	**0.20 [−0.03, 0.61] _b_**	
PMT (PR) →PFB (YR)	**−0.03 [−0.10, 0.002] _b_**	0.02 [−0.01, 0.10]	0.02 [−0.04, 0.13]	−0.01 [−0.14, 0.03]	0.003 [−0.04, 0.08]	−0.003 [−0.07, 0.04]	**−0.09 [−0.28, −0.01] _a_**	0.08 [−0.02, 0.26]
PMT (PR) →PMT (YR)	**−0.03 [−0.09, −0.01] _a_**		−0.02 [−0.10, 0.01]		−0.02 [−0.07, 0.004]		−0.01 [−0.06, 0.04]	
Total Indirect	**−0.17 [−0.46, 0.03] _b_**	−0.12 [−0.40, 0.06]	**−0.46 [−1.01, −0.10] _a_**	0.09 [−0.31, 0.52]	**−0.38 [−0.85, −0.09] _a_**	**0.30 [0.03, 0.69] _a_**	0.04 [−0.25, 0.42]	0.21 [−0.08, 0.65]
**N Cohesion →**								
CSEgal Beliefs (PR)	0.01 [−0.03, 0.04]		−0.02 [−0.08, 0.02]		−0.01 [−0.05, 0.02]		**−0.04 [−0.10, 0.01] _b_**	
PFB Beliefs (PR)	0.02 [−0.01, 0.08]		−0.03 [−0.10, 0.03]	**0.06 [0.001, 0.15] _a_**	0.02 [−0.02, 0.07]		**0.05 [0.01, 0.14] _a_**	
PMT Beliefs (PR)	−0.01 [−0.03, 0.003]		−0.02 [−0.06, 0.004]	0.01 [−0.01, 0.04]	−0.02 [−0.06, 0.003]	0.02 [−0.003, 0.05]	0.01 [−0.003, 0.04]	
CSEgal (PR)→ CSEgal (YR)	**−0.01 [−0.02, −0.001] _a_**		**−0.02 [−0.07, −0.004] _a_**	0.01 [−0.004, 0.03]	**−0.02 [−0.04, −0.003] _a_**	0.01 [−0.001, 0.03]	−0.01 [−0.02, 0.00]	
CSEgal (PR)→ PFB (YR)	0.004 [0.00, 0.02]		−0.002 [−0.02, 0.01]		0.00 [−0.01, 0.01]		**0.01 [0.00, 0.04] _b_**	
PFB (PR) → CSEgal (YR)	**0.02 [0.004, 0.05] _a_**	−0.001 [−0.01, 0.01]	**0.07 [0.03, 0.16] _a_**	0.002 [−0.01, 0.03]	**0.05 [0.02, 0.11**] _a_	0.002 [−0.001, 0.02]	**0.02 [−0.002, 0.06] _b_**	−0.001 [−0.02, 0.01]
PFB (PR) →PFB (YR)	−0.01 [−0.02, 0.00]		0.003 [−0.01, 0.02]		0.001 [−0.01, 0.02]		**−0.02 [−0.06, −0.003] _a_**	
Total Indirect	**0.03 [0.002, 0.08] _a_**	0.02 [−0.01, 0.05]	−0.02 [−0.08, 0.04]	**0.06 [0.01, 0.13] _a_**	0.02 [−0.03, 0.08]	0.03 [−0.01, 0.09]	0.03 [−0.01, 0.09]	0.02 [−0.03, 0.07]
**P Discrimination →**								
PMT Beliefs (PR) →	−0.01 [−0.04, 0.003]		**−0.03 [−0.09, 0.00] _b_**	0.01 [−0.01, 0.06]	**−0.03 [−0.08, −0.001] _a_**	**0.03 [0.001, 0.08] _a_**	0.02 [−0.003, 0.07]	
Total Indirect	−0.02 [−0.06, 0.02]	−0.01 [−0.05, 0.02]	−0.02 [−0.08, 0.04]	0.02 [−0.04, 0.08]	−0.02 [0.08, 0.03]	0.03 [−0.02, 0.08]	0.02 [−0.03, 0.07]	0.03 [−0.01, 0.09]
**P ERI →**								
CSEgal (PR) → CSEgal (YR)	−0.003 [−0.01, 0.00]		**−0.01 [−0.04, 0.00] _b_**	0.003 [−0.001, 0.02]	**−0.01 [−0.03, 0.00] _b_**	0.003 [0.00, 0.02]	−0.002 [−0.01, 0.00]	

Note. Bolded estimates are significant. Subscripts denote statistical significance based on confident intervals. a = statistically significant (95% CI does not include 0), b = marginally significant (90% CI does not include 0). CSEgal = Cultural-Egalitarianism, PFB = Preparation for Bias, PMT = Promotion of Mistrust; N Disadvantage = Neighborhood Disadvantage, N Cohesion = Neighborhood Cohesion, N Diversity = Neighborhood Diversity; P Discrimination = Parental Discrimination; P ERI = Parental Ethnic-Racial Identity, PR = Parent-Report, YR = Youth Report.

## Data Availability

The data presented in this study are available upon request from the corresponding author due to privacy restrictions expected by the participants when they agreed to participate in this study.

## Notes

1 The term parents is used inclusively to refer to any primary caregiver responsible for the daily parenting and care of the target child. This may include, but is not limited to, biological parents, adoptive parents, stepparents, or other guardians serving in a parental role.

2 All measures have been validated or shown to be reliable among Black and Latino populations.

## Supplementary Materials

The following supporting information can be downloaded at: https://www.mdpi.com/article/10.3390/bs15111437/s1, Table S1. Unstandardized estimates of direct effects and z-scores for parameter differences. Table S2. Standardized Indirect, Total Indirect, and Total Effects and 95% Confidence Intervals.
