# Supplementary material for: The Contextualized Impact of Ethnic-Racial Socialization on Black and Latino Youth’s Self-Esteem and Ethnic-Racial Identity
Source: Behav Sci (Basel). 2025 Oct 22;15(11):1437. doi: 10.3390/bs15111437 (PMC12649170; doi:10.3390/bs15111437)
Supplement: Supplementary file 1 [file behavsci-15-01437-s001.zip › behavsci-3729405-supplementary.pdf]

Supplemental Table S1. Unstandardized estimates of direct effects and z-scores for parameter differences

Variables	Model 1: Self-Esteem			Model 2: Centrality			Model 3: Private Regard			Model 4: Public Regard		
	Latino	Black	z	Latino	Black	z	Latino	Black	z	Latino	Black	z
	B (SE)	B (SE)		B (SE)	B (SE)		B (SE)	B (SE)		B (SE)	B (SE)	
**Outcome**												
N Disadvantage →	-.20** (.07)	-0.10 (0.11)	-0.77	-.27* (.11)	0.18+ (0.10)	-3.06**	-0.24* (0.08)	0.14 (0.09)	-2.75*	-0.16 (0.10)	-0.22 (0.14)	0.40
N Diversity →	1.77* (.85)	-0.07 (0.59)	1.80+	1.34 (.95)	1.14 (0.85)	0.16	0.49 (0.75)	-0.15 (0.62)	0.67	0.09 (0.90)	-0.05 (0.66)	0.12
N Cohesion →	-.12* (.06)	0.02 (0.12)	-1.11	-.05 (.10)	0.08 (0.15)	-0.73	0.08 (0.08)	0.03 (0.11)	0.35	-0.01 (0.09)	-0.26+ (0.14)	1.45
P Discrimination →	-.02 (.07)	0.09 (0.11)	-0.82	.15 (.10)	0.08 (0.12)	0.50	0.09 (0.07)	0.03 (0.01)	0.56	-0.01 (0.09)	-0.52*** (0.15)	2.94***
P Ethnic-Racial Identity →	-.04 (.08)	0.04 (0.11)	-0.61	.01 (.09)	0.04 (0.12)	-0.21	-0.001 (0.07)	-0.02 (0.09)	0.15	0.15+ (0.08)	0.24 (0.15)	-0.53
CSEgal Beliefs (PR) →	.15 (.15)	-0.08 (0.24)	0.82	-.03 (.20)	-0.20 (0.27)	0.50	-0.03 (0.17)	-0.09 (0.20)	0.24	-0.11 (0.22)	-0.41 (0.24)	0.85
PFB Beliefs (PR) →	.00 (.14)	0.17 (0.17)	-0.85	-.18 (.20)	0.31 (0.22)	-1.65+	0.01 (0.17)	0.17 (0.19)	-0.64	0.21 (0.17)	0.50* (0.24)	-0.96
PMT Beliefs (PR) →	-.10^{+} (.06)	0.01 (0.11)	-0.76	-.19* (.09)	0.11 (0.11)	-2.15*	-0.15* (0.07)	0.20** (0.07)	-3.37***	0.08 (0.10)	0.12 (0.13)	-0.24
CSEgal (YR) →	.25+ (.14)	0.13 (0.27)	0.38	.84*** (.21)	-0.26 (0.28)	3.12**	0.61*** (0.16)	-0.23 (0.26)	2.79*	0.09 (0.18)	0.45 (0.31)	-0.99
PFB (YR) →	-.12 (.11)	0.01 (0.19)	-0.60	.00 (.21)	0.20 (0.23)	-0.64	-0.10 (0.15)	0.23 (0.17)	-1.44	-0.49** (0.19)	-0.32 (0.29)	-0.49
PMT (YR) →	-.24 (.14)	-0.24 (0.25)	0.00	-.05 (.26)	-0.18 (0.28)	0.33	-0.09 (0.19)	-0.12 (0.22)	0.13	0.04 (0.26)	-0.07 (0.34)	0.25
**CSEgal (YR)**												
CS-Egal Beliefs (PR) →	-0.13 (0.10)	-0.24 (0.14)	0.65	-.12 (.10)	-0.24 (0.15)	0.67	-.12 (.10)	-0.23 (0.15)	0.59	-0.13 (0.10)	-0.23 (0.14)	0.57
PFB Beliefs (PR) →	0.45*** (0.10)	0.01 (0.14)	2.61**	.45*** (.10)	0.01 (0.14)	2.55*	.45*** (.10)	0.01 (0.14)	2.55*	0.45*** (0.10)	0.00 (0.14)	2.67**
PMT Beliefs (PR) →	-0.08 (0.05)	0.07 (0.09)	-1.33	-.08 (.05)	0.08 (0.09)	-1.46	-.08 (.05)	0.07 (0.09)	-1.40	-0.08 (0.05)	0.06 (0.09)	-1.26
**PFB (YR)**												
CSEgal Beliefs (PR) →	-0.20 (0.13)	-0.11 (0.19)	-0.39	-.20 (.13)	-0.12 (0.19)	-0.37	-.20 (.13)	-0.13 (0.20)	-0.32	-.21 (.13)	-0.13 (0.19)	-0.35
PFB Beliefs (PR) →	0.30** (0.11)	0.05 (0.17)	1.23	.30** (.11)	0.05 (0.17)	1.19	.30** (.11)	0.06 (0.17)	1.15	.29** (.11)	0.06 (0.17)	1.14
PMT Beliefs (PR) →	0.09 (0.06)	-0.09 (0.07)	1.88+	.09 (.06)	-0.09 (0.07)	1.90+	.09 (.06)	-0.08 (0.07)	1.87+	.09 (.06)	-0.08 (0.07)	1.79+
**PMT (YR)**												
CSEgal Beliefs (PR) →	-0.04 (0.10)	-0.08 (0.17)	0.23	-0.04 (.10)	-0.09 (0.17)	0.24	-0.04 (.10)	-0.09 (0.17)	0.24	-0.04 (.10)	-0.09 (0.17)	0.31
PFB Beliefs (PR) →	-0.04 (0.09)	-0.18 (0.16)	0.76	-.04 (.09)	-0.19 (0.16)	0.81	-.04 (.09)	-0.18 (0.16)	0.84	-.04 (.09)	-0.17 (0.16)	0.72
PMT Beliefs (PR) →	0.07** (0.03)	0.01 (0.07)	0.76	0.07** (.03)	0.01 (0.08)	0.77	0.07** (.03)	0.01 (0.08)	0.77	0.07** (.03)	0.01 (0.08)	0.71
**CSEgal Beliefs (PR)**												
N Disadvantage →	-0.01 (0.08)	-0.09 (0.08)	0.67	-0.01 (0.08)	-0.08 (0.08)	0.66	-0.01 (0.08)	-0.08 (0.07)	0.66	-0.01 (0.08)	-0.09 (0.08)	0.67
N Diversity →	0.61 (0.63)	0.18 (0.38)	0.59	0.61 (0.63)	0.18 (0.38)	0.59	0.61 (0.63)	0.18 (0.38)	0.59	0.64 (0.64)	0.19 (0.38)	0.61
N Cohesion →	0.18** (0.06)	0.14* (0.07)	0.48	0.18** (0.06)	0.14* (0.07)	0.47	0.18** (0.06)	0.14* (0.07)	0.47	0.18** (0.06)	0.14* (0.07)	0.46
P Discrimination →	-0.13 (0.10)	0.07 (0.07)	-1.56	-0.13 (0.10)	0.07 (0.07)	-1.55	-0.13 (0.10)	0.07 (0.07)	-1.55	-0.13 (0.10)	0.07 (0.07)	-1.54
P Ethnic-Racial Identity →	0.08 (0.08)	0.07 (0.07)	0.01	0.08 (0.08)	0.07 (0.07)	0.01	0.08 (0.08)	0.07 (0.07)	0.01	0.08 (0.08)	0.07 (0.07)	0.08
**PFB Beliefs (PR)**												
N Disadvantage →	-0.12 (0.07)	-0.08 (0.08)	-0.03	-0.12 (0.07)	-0.08 (0.08)	-0.04	-0.12 (0.07)	-0.08 (0.08)	-0.04	-0.12 (0.07)	-0.08 (0.08)	-0.03
N Diversity →	0.24 (0.56)	-0.05 (0.46)	-0.19	0.24 (0.56)	-0.04 (0.46)	-0.19	0.24 (0.56)	-0.04 (0.46)	-0.19	0.26 (0.57)	-0.05 (0.46)	-0.18
N Cohesion →	0.17* (0.07)	0.24** (0.08)	-0.11	0.17* (0.07)	0.24** (0.08)	-0.12	0.17* (0.07)	0.24** (0.08)	-0.15	0.17* (0.07)	0.24** (0.08)	-0.16
P Discrimination →	-0.05 (0.11)	0.10 (0.11)	-0.84	-0.05 (0.11)	0.10 (0.11)	-0.84	-0.05 (0.11)	0.10 (0.11)	-0.84	-0.05 (0.11)	0.09 (0.11)	-0.83
P Ethnic-Racial Identity →	0.07 (.08)	0.01 (0.07)	0.62	0.07 (.08)	0.01 (0.07)	0.62	0.07 (.08)	0.01 (0.07)	0.62	0.07 (.08)	0.01 (0.07)	0.65
**PMT Beliefs (PR)**												
N Disadvantage →	-0.27* (0.13)	-0.26* (0.13)	-0.32	-0.27* (0.13)	-0.26* (0.13)	-0.35	-0.27* (0.13)	-0.26* (0.13)	-0.36	-0.27* (0.13)	-0.26* (0.13)	-0.34
N Diversity →	1.85+ (1.11)	2.11* (0.84)	0.40	1.85+ (1.11)	2.11* (0.84)	0.39	1.85+ (1.10)	2.11* (0.84)	0.39	1.85+ (1.12)	2.09* (0.84)	0.43
N Cohesion →	0.10 (0.09)	0.12 (0.14)	-0.61	0.10 (0.09)	0.12 (0.14)	-0.64	0.10 (0.09)	0.12 (0.14)	-0.64	0.10 (0.09)	0.12 (0.14)	-0.67
P Discrimination →	0.10 (0.12)	0.25+ (0.14)	-0.97	0.10 (0.12)	0.25+ (0.14)	-0.96	0.10 (0.12)	0.25+ (0.14)	-0.96	0.10 (0.12)	0.25+ (0.14)	-0.94
P Ethnic-Racial Identity →	-0.02 (0.10)	-0.12 (0.12)	0.59	-0.02 (0.10)	-0.12 (0.12)	0.60	-0.02 (0.10)	-0.12 (0.12)	0.60	-0.02 (0.10)	-0.13 (0.12)	0.64

Note: N = 184. Table displays undstandardized path coefficients (B), standard errors (SE), and z-scores for group differences between Latino and Black families across four outcomes. *p < .05; **p < .01; ***p < .001.
N Disadvantage = Neighborhood disadvantage; N Diversity = Neighborhood Diversity; P Discrimination = Parental Discrimination, P Ethnic-Racial Identity = Parental Ethnic-Racial Identity
CSEgal= CSEgal; PFB = Preparation for Bias , PMT = PMT ; PR = Parent Report; YR = Youth Report.

Supplemental Table S2: Standardized Indirect, Total Indirect, and Total Effects and 95% Confidence Intervals

Model 1: Self-Esteem

Mediator	Latino	Black	Latino	Black	Latino	Black	Latino	Black	Latino	Black
	B [95% CI]		B [95% CI]		B [95% CI]		B [95% CI]			B [95% CI]
	**Neighborhood Disadvantage**		**Neighborhood Diversity**		**Neighborhood Cohesion**		**Parental Discrimination**		**Parental ERI**	
CSEgal Beliefs (PR)	-.002 [-.04, .01]		.02 [-.05, .21]		.01 [-.03, .04]		-.002 [-.04, .01]		.003 [-.01, .03]	
PFB Beliefs (PR)	-.01 [-.06, .004]		.002 [-.07, .13]		.02 [-.01, .08]		.00 [-.02, .02]		.01 [-.004, .04]	
PMT Beliefs (PR)	.02 [-.003, .06]		-.12 [-.36, .03]		-.01 [-.03, .003]		-.01 [-.04, .003]		.003 [-.004, .02]	
CSEgal (PR) →CSEgal (YR)	.002 [-.001, .01]		-.01 [-.08, .003]		**-.01 [-.02, -.001]$_a$**		.002 [-.001, .02]		-.003 [-.01, .00]	
CSEgal (PR) → FB (YR)	-.001 [-.01, .00]		.01 [-.003, .06]		.004 [.00, .02]		-.001 [-.01, .001]		.002 [.00, .01]	
CSEgal (PR) → PMT (YR)	-.001 [-.01, .001]		.004 [-.01, .05]		.002 [-.004, .01]		-.001 [-.01, .001]		.001 [-.001, .01]	
PFB (PR) →CSEgal (YR)	**-.01 [-.04, -.001]$_a$**	.001 [-.002, .10]	.002 [-.06, .09]	.00 [-.02, .01]	**.02 [.004, .05]$_a$**	-.001 [-.01, .01]	.00 [-.02, .02]	.00 [-.004, .003]	.01 [-.003, .03]	.00 [-.01, .001]
PFB (PR) → PFB (YR)	.003 [.00, .02]		.00 [-.03, .02]		-.01 [-.02, .00]		.00 [-.01, .004]		-.001 [-.01, .00]	
PFB (PR) → PMT (YR)	-.002 [-.02, .00]		.00 [-.02, .04]		.004 [-.002, .02]		.00 [-.004, .01]		.001 [-.001, .01]	
PMT (PR) → CSEgal (YR)	**.001 [-.003, .01]**		-.01 [-..8, .02]		.00 [-.01, .001]		-.001 [-.01, .002]		.00 [-.001, .01]	
PMT (PR) → PFB (YR)	.004 [.00, .02]	-.003 [-.02, .001]	**-.03+ [-.10, .002]$_b$**	.02 [-.01, .10]	-.001 [-.01, .00]	.01 [.00, .01]	-.002 [-.01, .00]	.002 [-.00, .01]	.001 [.00, .01]	-.001 [-.01, .001]
PMT (PR) → PMT (YR)	**.004 [.001, .02]$_a$**		**-.03 [-.09, -.01]$_a$**		-.002 [-.01, .00]		-.003 [-.01, .00]		.001 [-.001, .01]	
Total Indirect	.004 [-.03, .05]	.01 [-.03, .05]	**-.17+ [-.46, .03]$_b$**	-.12 [-.40, .06]	**.03 [.002, .08]$_a$**	.02 [-.01, .05]	-.02 [-.06, .02]	-.01 [-.05, .02]	.02 [-.01, .12]	.01 [-.01, .04]
Total	**-.16 [-.27, -.05]$_a$**	**-.16 [-.26, -.05]$_a$**	1.60 [.17, 3.22]	.144 [-.79, 1.12]	-.05 [-.15, .05]	-.06 [-.17, .03]	-.02 [-.13, .09]	-.01 [-.13, .09]	-.001 [-.13, .12]	-.01 [-.13, .15]

Mediator	Latino	Black	Latino	Black	Latino	Black
	B [95% CI]		B [95% CI]			B [95% CI]
	**CSEgal Beliefs (PR)**		**PFB Beliefs (PR)**		**PMT Beliefs (PR)**	
CSEgal (YR)	**-.04 [-.12, -.002]$_a$**		**.09 [.01, .19]$_a$**	-.01 [-.06, .03]	-.01 [-.04, .01]	
PFB (YR)	.02 [-.01, .08]		**-.03+ [-.09, .003]$_b$**		-.01 [-.05, .003]	.01 [-.004, .05]
PMT (YR)	.01 [-.03, .06]		.02 [-.01, .09]		**-.02 [-.04, -.003]$_a$**	
Total Indirect	-.01 [-.09, .07]		.09 [-.02, .22]	-.01 [-.10, .08]	**-.03 [-.08, -.01]$_a$**	-.01 [-.05, .03]
Total	.04 [-.17, .27]		**.19 [.02, .38]$_a$**	.09 [-.11, .29]	**-.10+ [-.19, .00]$_b$**	-.07 [-.17, .03]

Model 2: Centrality

Mediator	Latino	Black	Latino	Black	Latino	Black	Latino	Black	Latino	Black
	B [95% CI]		B [95% CI]		B [95% CI]		B [95% CI]			B [95% CI]
	**Neighborhood Disadvantage**		**Neighborhood Diversity**		**Neighborhood Cohesion**		**Parental Discrimination**		**Parental ERI**	
CSEgal Beliefs (PR)	.01 [-.01, .06]		-.04 [-.33, .04]		-.02 [-.08, .02]		.01 [-.01, .06]		-.01 [-.06, .01]	
PFB Beliefs (PR)	.02 [-.01, .08]	**-.03 [-.11, -.002]$_a$**	-.004 [-.22, .13]	.01 [-.21, .30]	-.03 [-.10, .03]	**.06 [.001, .15]$_a$**	.00 [-.04, .03]	.00 [-.05, .06]	-.01 [-.06, .01]	.02 [-.01, .07]
PMT Beliefs (PR)	**.05 [.01, .13]$_a$**	-.02 [-.09, .02]	**-.33 [-.80, -.04]$_a$**	.14 [-.13, .55]	-.02 [-.06, .004]	.01 [-.01, .04]	**-.03+ [-.09, .00]$_b$**	.01 [-.01, .06]	.01 [-.01, .05]	-.004 [-.03, .01]
CSEgal (PR) →CSEgal (YR)	.01 [-.003, .04]	-.002 [-.02, .001]	-.05 [-.22, .02]	.01 [-.01, .12]	**-.02 [-.07, -.004]$_a$**	.01 [-.004, .03]	.01 [-.01, .04]	-.002 [-.02, .002]	**-.01+ [-.04, .00]$_b$**	.003 [-.001, .02]
CSEgal (PR) → FB (YR)	.001 [-.001, .02]		-.004 [-.08, .01]		-.002 [-.02, .01]		.001 [-.002, .01]		-.001 [-.01, .002]	
CSEgal (PR) → PMT (YR)	.00 [-.01, .00]		.002 [-.003, .06]		.001 [-.002, .01]		.00 [-.01, .001]		.001 [-.001, .01]	
PFB (PR) →CSEgal (YR)	**-.04 [-.12, -.01]$_a$**	-.001 [-.02, .002]	.01 [-.22, .27]	.00 [-.02, .04]	**.07 [.03, .16]$_a$**	.002 [-.01, .03]	.00 [-.05, .06]	.00 [-.01, .01]	.02 [-.01, .08]	.00 [-.001, .01]

Mediator										
PFB (PR) → PFB (YR)	-.002 [-.02, .002]		.00 [-.02, .04]		.003 [-.01, .02]		.00 [-.01, .01]		.001 [-.001, .01]	
PFB (PR) → PMT (YR)	-.001 [-.02, .001]		.00 [-.01, .03]		.002 [-.002, .02]		.00 [-.003, .01]		.001 [-.001, .01]	
PMT (PR) → CSEgal (YR)	.01 [-.01, .04]	-.002 [-.02, .004]	-.04 [-.23, .09]	.01 [-.03, .13]	-.002 [-.02, .004]	.001 [-.001, .01]	-.003 [-.03, .01]	.001 [-.002, .01]	.001 [-.002, .02]	.00 [-.01, .001]
PMT (PR) → PFB (YR)	-.002 [-.02, .01]	.002 [-.004, .02]	.02 [-.04, .13]	-.01 [-.14, .03]	.001 [-.002, .01]	-.001 [-.01, .001]	.001 [-.003, .02]	-.001 [-.02, .002]	.00 [-.01, .001]	.00 [-.01, .01]
PMT (PR) → PMT (YR)	.003 [-.001, .02]		-.02 [-.10, .01]		-.001 [-.01, .00]		-.002 [-.01, .001]		.00 [.00, .01]	
Total Indirect	.05 [-.01, .13]	**-.05 [-.13, -.002]**$_a$	**-.46 [-1.01, -.10]**$_a$	.09 [-.31, .52]	-.02 [-.08, .04]	**.06 [.01, .13]**$_a$	-.02 [-.08, .04]	.02 [-.04, .08]	.002 [-.05, .05]	.01 [-.03, .05]
Total	-.22 [-.41, -.03]$_a$	.13 [-.05, .34]	.84 [-.36, 2.06]	**1.39 [.36, 2.51]**$_a$	-.01 [-.17, .14]	.06 [-.09, .22]	.10 [-.05, .23]	.13 [-.01, .27]	.02 [-.11, .14]	.03 [-.10, .15]

Mediator	Latino B [95% CI]	Black	Latino B [95% CI]	Black	Latino B [95% CI]	Black
	**CSEgal Beliefs (PR)**		**PFB Beliefs (PR)**		**PMT Beliefs (PR)**	
CSEgal (YR)	**-.14 [-.33, -.02]**$_a$	.04 [-.03, .16]	**.36 [.17, .65]**$_a$	.01 [-.04, .12]	-.02 [-.11, .05]	.01 [-.02, .06]
PFB (YR)	-.01 [-.11, .03]		.02 [-.04, .11]		.01 [-.02, .06]	-.01 [-.06, .02]
PMT (YR)	.01 [-.01, .09]		.01 [-.01, .11]		-.01 [-.04, .01]	
Total Indirect	**-.14+ [-.33, .00]**$_b$	.04 [-.07, .18]	**.38 [.19, .67]**$_a$	.04 [-.07, .17]	-.02 [-.12, .07]	-.01 [-.07, .04]
Total	-.27 [-.61, .06]	-.10 [-.37, .20]	.24 [-.09, .60]	**.34 [.03, .65]**$_a$	**-.20 [-.37, -.01]**$_a$	.06 [-.11, .22]

Model 3: Private Regard

Mediator	Latino B [95% CI]	Black	Latino B [95% CI]	Black	Latino B [95% CI]	Black	Latino B [95% CI]	Black	Latino	Black B [95% CI]
	**Neighborhood Disadvantage**		**Neighborhood Diversity**		**Neighborhood Cohesion**		**Parental Discrimination**		**Parental ERI**	
CSEgal Beliefs (PR)	.003 [-.01, .04]		-.02 [-.21, .04]		-.01 [-.05, .02]		.003 [-.01, .04]		-.01 [-.04, .01]	
PFB Beliefs (PR)	-.01 [-.06, .01]		.002 [-.07, .13]		.02 [-.02, .07]		.00 [-.02, .02]		.004 [-.01, .04]	
PMT Beliefs (PR)	**.05 [.01, .12]**$_a$	**-.05 [-.12, -.01]**$_a$	**-.30 [-.69, -.07]**$_a$	**.31 [.07, .72]**$_a$	-.02 [-.06, .003]	.02 [-.003, .05]	**-.03 [-.08, -.001]**$_a$	**.03 [.001, .08]**$_a$	.01 [-.01, .04]	-.01 [-.04, .01]
CSEgal (PR) → CSEgal (YR)	.01 [-.002, .03]	-.002 [-.02, .001]	-.03 [-.15, .01]	.01 [-.004, .12]	**-.02 [-.04, -.003]**$_a$	.01 [-.001, .03]	.004 [-.01, .03]	-.002 [-.02, .002]	**-.01 [-.03, .00]**$_b$	.003 [.00, .02]
CSEgal (PR) → FB (YR)	.00 [-.002, .01]		-.001 [-.04, .01]		.00 [-.01, .01]		.00 [-.002, .01]		.00 [-.01, .002]	
CSEgal (PR) → PMT (YR)	.00 [-.01, .00]		.002 [-.003, .05]		.001 [-.002, .01]		.00 [-.01, .001]		.00 [-.001, .01]	
PFB (PR) → CSEgal (YR)	**-.03 [-.08, -.01]**$_a$	-.001 [-.02, .002]	.01 [-.15, .19]	.00 [-.02, .04]	**.05 [.02, .11]**$_a$	.002 [-.001, .02]	.00 [-.04, .04]	.00 [-.004, .01]	.01 [-.01, .05]	.00 [-.001, .01]
PFB (PR) → PFB (YR)	.00 [-.01, .004]		.00 [-.02, .02]		.001 [-.01, .02]		.00 [-.004, .004]		.00 [-.002, .01]	
PFB (PR) → PMT (YR)	-.001 [-.01, .00]		.00 [-.01, .03]		.002 [-.001, .01]		.00 [-.002, .004]		.001 [.000, .01]	
PMT (PR) → CSEgal (YR)	.004 [-.01, .03]	-.002 [-.02, .003]	-.03 [-.17, .07]	.01 [-.02, .13]	-.001 [-.01, .003]	.001 [-.001, .01]	-.002 [-.02, .01]	.001 [-.002, .01]	.001 [-.002, .01]	.00 [-.01, .001]
PMT (PR) → PFB (YR)	.00 [-.01, .01]	.00 [-.01, .01]	.003 [-.04, .08]	-.003 [-.07, .04]	.00 [-.002, .01]	.00 [-.01, .002]	.00 [-.004, .01]	.00 [-.01, .003]	.00 [-.004, .001]	.00 [-.001, .004]
PMT (PR) → PMT (YR)	.002 [.00, .01]		-.02 [-.07, .004]		-.001 [-.01, .00]		-.001 [-.01, .00]		.00 [.00, .01]	

Mediator	Latino	Black	Latino	Black	Latino	Black	Latino	Black	Latino	Black
Total Indirect	.02 [-.03, .09]	**-.06 [-.13, -.02]**$_a$	**-.38 [-.85, -.09]**$_a$	**.30 [.03, .69]**$_a$	.02 [-.03, .08]	.03 [-.01, .09]	-.02 [.08, .03]	.03 [-.02, .08]	.02 [-.02, .06]	-.004 [-.04, .03]
Total	**-.17 [-.31, -.03]**$_a$	.08 [-.01, .25]	-.27 [-1.08, .58]	.40 [-.33, 1.23]	.08 [-.03, .21]	.09 [-.02, .21]	.04 [-.07, .15]	**.09 [-.004, .19]**$_a$	-.01 [-.12, .09]	-.03 [-.13, .06]

	Latino	Black	Latino	Black	Latino	Black
Mediator	B [95% CI]		B [95% CI]		B [95% CI]	
	**CSEgal Beliefs (PR)**		**PFB Beliefs (PR)**		**PMT Beliefs (PR)**	
CSEgal (YR)	**-.09 [-.22, -.01]**$_a$	.04 [-.01, .16]	**.25 [.12, .44]**$_a$	.01 [-.03, .11]	-.01 [-.08, .04]	.01 [-.02, .06]
PFB (YR)	-.003 [-.07, .04]		.003 [-.04, .07]		.002 [-.02, .04]	-.001 [-.04, .02]
PMT (YR)	.01 [-.01, .06]		.01 [-.01, .07]		-.01 [-.03, .003]	
Total Indirect	**-.09+ [-.22, .01]**$_b$	.05 [-.03, .17]	**.26 [.13, .45]**$_a$	.02 [-.06, .13]	-.02 [-.09, .04]	-.003 [-.05, .04]
Total	-.15 [-.42, .10]	-.02 [-.23, .21]	**.34 [.11, .59]**$_a$	.11 [-.11, .31]	**-.18 [-.32, -.05]a**	.16 [.03, .29]

Model 4: Public Regard

	Latino	Black	Latino	Black	Latino	Black	Latino	Black	Latino	Black
Mediator	B [95% CI]		B [95% CI]		B [95% CI]		B [95% CI]		B [95% CI]	
	**Neighborhood Disadvantage**		**Neighborhood Diversity**		**Neighborhood Cohesion**		**Parental Discrimination**		**Parental ERI**	
CSEgal Beliefs (PR)	.01 [-.01, .07]		-.08 [-.43, .04]		**-.04+ [-.10, .01]**$_b$		.01 [-.01, .08]		-.02 [-.07, .004]	
PFB Beliefs (PR)	**-.03 [-.10, -.004]**$_a$		.01 [-.18, .25]		**.05 [.01, .14]**$_a$		.00 [-.04, .05]		.01 [-.01, .06]	
PMT Beliefs (PR)	**-.03+ [-.10, .003]**$_b$		**.20+ [-.03, .61]**$_b$		.01 [-.003, .04]		.02 [-.003, .07]		-.01 [.04, .01]	
CSEgal (PR) →CSEgal (YR)	.002 [-.001, .02]		-.01 [-.08, .004]		-.01 [-.02, .00]		.002 [-.001, .02]		-.002 [-.01, .00]	
CSEgal (PR) → FB (YR)	-.004 [-.03, .002]		.03 [-.01, .15]		**.01+ [.00, .04]**$_b$		-.004 [-.03, .004]		.01 [-.001, .03]	
CSEgal (PR) → PMT (YR)	.00 [-.01, .001]		.001 [-.01, .04]		.00 [-.004, .01]		.00 [-.01, .001]		.00 [-.001, .01]	
PFB (PR) →CSEgal (YR)	**-.01+ [-.04, .00]**$_b$	.001 [-.002, .01]	.002 [-.06, .10]	.00 [-.02, .01]	**.02+ [-.002, .06]**$_b$	-.001 [-.02, .01]	.00 [-.02, .02]	.00 [-.04, .04]	.004 [-.002, .03]	.00 [-.01, .001]
PFB (PR) → PFB (YR)	**.01 [.001, .04]**$_a$		-.002 [-.09, .06]		**-.02 [-.06, -.003]**$_a$		.00 [-.02, .01]		-.01 [-.03, .002]	
PFB (PR) → PMT (YR)	.00 [-.01, .002]		.00 [-.01, .02]		.001 [-.01, .01]		.00 [-.003, .004]		.00 [-.001, .01]	
PMT (PR) → CSEgal (YR)	.002 [-.002, .02]		-.01 [-.09, .02]		-.001 [-.01, .001]		-.001 [-.01, .001]		.00 [.00, .01]	
PMT (PR) → PFB (YR)	**.01 [.002, .04]**$_a$	-.01 [-.04, .002]	**-.09 [-.28, -.01]**$_a$	.08 [-.02, .26]	-.01 [-.02, .001]	.004 [-.02, .02]	-.01 [-.03, .00]	.01 [-.002, .03]	.002 [-.002, .02]	-.002 [-.02, .002]
PMT (PR) → PMT (YR)	.001 [-.01, .01]		-.01 [-.06, .04]		.00 [-.01, .002]		.00 [-.01, .004]		.00 [-.001, .004]	
Total Indirect	-.04 [-.10, .01]	**-.05 [-.12, -.01]**$_a$	.04 [-.25, .42]	.21 [-.08, .65]	.03 [-.01, .09]	.02 [-.03, .07]	.02 [-.03, .07]	.03 [-.01, .09]	.00 [-.04, .04]	-.01 [-.05, .02]
Total	**-.19 [-.34, -.04]**$_a$	**-.21 [-.36, -.06]**$_a$	.02 [-.95, .95]	.19 [-.81, 1.13]	-.05 [-.20, .10]	-.05 [-.22, .09]	-.004 [-.15, .16]	-.47 [-.70, -.22]	**.16 [.04, .29]**$_a$	**.15 [.03, .28]**$_a$

	**Latino**	**Black**	**Latino**	**Black**	**Latino**	**Black**
Mediator	B [95% CI]		B [95% CI]		B [95% CI]	
	**CSEgal Beliefs (PR)**		**PFB Beliefs (PR)**		**PMT Beliefs (PR)**	
CSEgal (YR)	-.04 [-.12, .01]		.09 [-.02, .24]	-.01 [-.08, .03]	-.01 [-.04, .01]	
PFB (YR)	**.08+ [-.01, .23]**$_b$		**-.09 [-.24, -.01]**$_a$		**-.05+ [-.12, .001]**$_b$	.04 [-.02, .12]
PMT (YR)	.002 [-.03, .07]		.004 [-.03, .07]		-.003 [-.03, .03]	

Total Indirect	.05 [-.07, .20]	.00 [-.16, .17]	-.10 [-.25, .02]	**-.06 [-.14, -.002]$_a$**	.03 [-.04, .11]
Total	**-.17 [-.50, .13]**	**.28 [.03, .55]$_a$**	**.19 [-.07, .46]**	**.05 [-.10, .21]**	**.14+ [-.01, .30]$_b$**

Note. Bolded estimates are significant. Subscripts denote statistical significance based on confident intervals. a = statistically significant (95% CI does not include 0), b = marginally significant (90% CI does not include 0). CSEgal = Cultural-Egalitarianism, PFB = Preparation for Bias, PMT = Promotion of Mistrust; ERI = Parental Ethnic-Racial Identity, PR = Parent-Report, YR = Youth Report.
